# Mechanisms and network pharmacological analysis of Yangyin Fuzheng Jiedu prescription in the treatment of hepatocellular carcinoma

**DOI:** 10.1002/cam4.5064

**Published:** 2022-08-31

**Authors:** Yuqing Xie, Fengna Yan, Xinhui Wang, Lihua Yu, Huiwen Yan, Qing Pu, Weihong Li, Zhiyun Yang

**Affiliations:** ^1^ Center of Integrative Medicine, Beijing Ditan Hospital Capital Medical University Beijing P.R. China; ^2^ School of Traditional Chinese Medicine Beijing University of Chinese Medicine Beijing P.R. China

**Keywords:** hepatocellular carcinoma, immunoregulation, network pharmacology, T cell exhaustion, tumor microenvironment, Yangyin Fuzheng Jiedu prescription

## Abstract

**Objective:**

To identify the key drugs of Yangyin Fuzheng Jiedu prescription (YFJP) and investigate their therapeutic effects against hepatocellular carcinoma (HCC) and the potential mechanism using network pharmacology.

**Methods:**

The H22 tumor‐bearing mouse model was established. Thirty male BALB/c mice were divided randomly into five groups. The mice were orally treated with either disassembled prescriptions of YFJP or saline solution continuously for 14 days. The mice were weighed every 2 days during treatment and the appearance of tumors was observed by photographing. The tumor inhibition rate and the spleen and thymus indexes were calculated. Hematoxylin and eosin and immunohistochemical staining were performed to observe the histological changes and tumor‐infiltrating lymphocytes. Cell apoptosis was determined by terminal deoxynucleotidyl transferase‐mediated dUTP nick‐end labeling staining. The proportion of CD8^+^ T cells and the expression of programmed cell death protein 1 (PD‐1), T cell immunoglobulin domain and mucin domain‐3 (Tim‐3), and T cell immunoreceptor with Ig and ITIM domains (TIGIT) were analyzed using flow cytometry. The production of serum cytokines was detected using the Milliplex® MAP mouse high sensitivity T cell panel kit. The active components of the key drugs and HCC‐related target proteins were obtained from the corresponding databases. The putative targets for HCC treatment were screened by target mapping, and potential active components were screened by constructing a component‐target network. The interactive targets of putative targets were obtained from the STRING database to construct the protein–protein interaction network. Gene ontology (GO) and Kyoto encyclopedia of genes and genomes pathway enrichment analyses were performed based on potential targets. The gene–gene inner and component‐target‐pathway networks were constructed and analyzed to screen the key targets. Western blotting was used to evaluate the protein expression of the key targets in the tumor‐bearing mouse model. The binding activity of the key targets and compounds was verified by molecular docking.

**Results:**

Among the three disassembled prescriptions of YFJP, the Fuzheng prescription (FZP) showed significant antitumor effects and inhibited weight loss during the treatment of H22 tumor‐bearing mice. FZP increased the immune organ index and the levels of CD8^+^ and CD3^+^ T cells in the spleen and peripheral blood of H22 tumor‐bearing mice. FZP also reduced the expression of PD‐1, TIGIT, and TIM3 in CD8^+^ T cells and the production of IL‐10, IL‐4, IL‐6, and IL‐1β. Network pharmacology and experimental validation showed that the key targets of FZP in the treatment of HCC were PIK3CA, TP53, MAPK1, MAPK3, and EGFR. The therapeutic effect on HCC was evaluated based on HCC‐related signaling pathways, including the PIK3‐Akt signaling pathway, PD‐L1 expression, and PD‐1 checkpoint pathway in cancer. GO enrichment analysis indicated that FZP positively regulated the molecular functions of transferases and kinases on the cell surface through membrane raft, membrane microarea, and other cell components to inhibit cell death and programmed cell death.

**Conclusion:**

FZP was found to be the key disassembled prescription of YFJP that exerted antitumor and immunoregulatory effects against HCC. FZP alleviated T cell exhaustion and improved the immunosuppressive microenvironment via HCC‐related targets, pathways, and biological processes.

## Graphical abstract

In vivo experiments were conducted to screen the key drugs of YFJP and explore its anti‐tumor mechanism. Network pharmacology, GO and KEGG enrichment analysis, molecular docking were conducted to further explore the potential mechanisms of the key drugs.

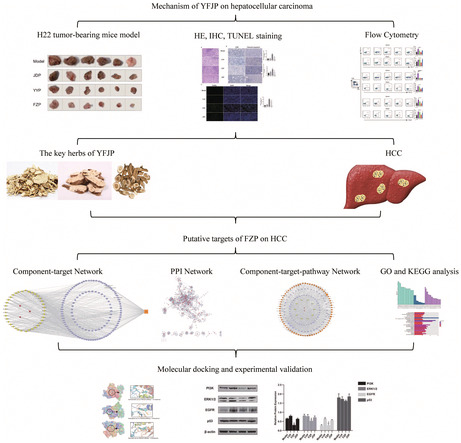

## 1 INTRODUCTION

Liver cancer is the sixth most common cancer worldwide, about 72% of which occurs in Asia, with China accounting for 47%. As a result, it is the fourth most common cause of cancer mortality worldwide and remains a significant contributor to the global cancer burden.^1^, ^2^ Hepatocellular carcinoma (HCC) is diagnosed in over 80% of cases of liver cancer.^3^ Conventional therapy for patients in the early and middle stages of HCC currently includes resection, local ablation, transplantation, and transarterial chemoembolization, while systemic therapy is used for patients in advanced stages. An alternative treatment using a combination of atezolizumab (anti‐PD‐L1 antibody) and bevacizumab (anti‐VEGF antibody) was approved by the US Food and Drug Administration (FDA) for advanced HCC. Before this, no treatment had surpassed the effect of sorafenib in the first line for more than a decade.^4^ However, due to the insidious onset of HCC, over 80% of the patients are not able to receive treatment at the time of diagnosis. Meanwhile, current treatment options for advanced stages of HCC lead to poor outcomes in most patients with HCC, with a 5‐year survival rate of 18%.^5^ Therefore, there is an urgent need to develop further therapeutic strategies.

T cells are considered to be critical for HCC immunotherapy, with cytotoxic T (CD8^+^) cells playing a key role in antitumor immunity. The tumor microenvironment (TME) is composed of multiple immunosuppressive cells, cytokines, chemokines, and the extracellular matrix (ECM). TME provides an environment for tumor genesis and development and is the key factor in tumor immune escape. Many studies have reported that CD8^+^ T cells that infiltrate into the HCC microenvironment are often characterized by the overexpression of inhibitory receptors and the progressive loss of effector function, defined as exhausted CD8^+^ T cells. Therefore, restoring exhausted CD8^+^ T cells and remodeling the immunosuppressive microenvironment is considered a promising therapeutic strategy for HCC.^6^, ^7^ Immune checkpoint inhibitors have been developed as a treatment option for advanced HCC, wherein the blockage of the inhibitory receptors can allow the immune response of cytotoxic T cells to emerge. An anti‐CTLA‐4 antibody (ipilimumab), an anti‐PD‐L1 antibody (atezolizumab), and two anti‐PD‐1 antibodies (nivolumab and pembrolizumab) have been approved by the FDA for combination therapy or monotherapy in patients with advanced HCC.^8^ However, adverse reactions have been reported from these treatments.^9^, ^10^ Several clinical studies have proved that traditional Chinese medicine (TCM) has a variety of synergistic effects on improving the incidence of HCC and the survival of patients with intermediate to advanced HCC.^11^, ^12^ To maximize the antitumor effects and minimize toxicity, novel combination strategies are being explored and developed within the field of TCM. As a result, the potential immunomodulatory effects of TCM have recently gained attention.

The immunomodulatory effects of TCM are exerted via multiple targets and pathways, which is consistent with its basic theoretical characteristics of holism and syndrome differentiation. Yangyin Fuzheng Jiedu prescription (YFJP) is a traditional Chinese herb decoction that has been clinically used in the treatment of HCC for many years. Preliminary clinical studies conducted by our team have confirmed the antitumor effect of YFJP on patients with HCC.^13^ Previous in vivo experiments showed that this prescription could relieve the exhaustion phenotype of CD8^+^ T cells and reduce the production of immunosuppressive cytokines in the TME to exert an antitumor effect.^14^ However, the key drugs of YFJP remain to be precisely identified.

Due to a large number of components in TCM prescriptions and the involvement of multiple molecular changes in complex diseases, such as HCC, elucidating the potential mechanisms of herbal formula alone by conventional experimental approaches is not sufficient.^15^ Network pharmacology is a scientific approach based on analyzing network data and systems biology,^16^ used to investigate the relationships among drugs, targets, components, and diseases by constructing biological networks. This allows revealing how the different components of TCM abolish the biological networks of diseases.

Network pharmacology has allowed for the transition from the current “one drug, one target” strategy to the “multi‐component network target” strategy.^17^ This approach is consistent with the key idea of holism within TCM theory. In recent years, network pharmacology has been increasingly applied to identify the active ingredients and therapeutic targets of traditional Chinese decoctions.

In this study, the traditional YFJP prescription was disassembled according to the TCM theory into Yangyin prescription (YYP), Fuzheng prescription (FZP), and Jiedu prescription (JDP) to explore the effects of the disassembled prescriptions on H22 tumor‐bearing mice. Herein, network pharmacology was used to investigate the mechanisms of the key dissembled prescriptions for the treatment of HCC. Molecular docking and in vivo experiments were used to verify the results. These results demonstrate that TCM can exert antitumor effects via multi‐component and multi‐target immune regulation.

## 2 MATERIALS AND METHODS

### 2.1 Preparation of experimental TCM

*Hedysarum multijugum Maxim*. (Chinese name: Huangqi, 20 g), *Atractylodes macrocephala Koidz*. (Chinese name: Baizhu, 9 g), Radix *Bupleuri* (Chinese name: Chaihu, 9 g), *Hedyotis diffusae Herba* (Chinese name: Baihua Shesehcao, 20 g), Radix *Sophorae flavescentis* (Chinese name: Kushen, 9 g), Radix *Cynanchi paniculati* (Chinese name: Xuchangqing, 12 g), Glehniae Radix (Chinese name: Beishashen, 15 g), and *Ophiopogon japonicus* (Chinese name: Maimendong, 15 g) were purchased from the Pharmacy of Traditional Chinese Medicine, Beijing Ditan Hospital. The disassembled prescriptions of YFJP were prepared using the following combinations: (i) *Hedysarum multijugum Maxim*. (20 g), *Atractylodes macrocephala Koidz*. (9 g), and Radix *Bupleuri* (9 g) for Fuzheng prescription (FZP); (ii) *Ophiopogon japonicus* (15 g) and *Glehniae* Radix (15 g) for Yangyin prescription (YYP); (iii) *Sophorae flavescentis* Radix (9 g), Hedyotis diffusae Herba (20 g), and Radix *Cynanchi paniculati* (12 g) for Jiedu prescription (JDP). Each drug combination was soaked in water (450 ml) for 20 min and decocted for 40 min. The liquids were then filtered into a beaker and boiled for 30 min with 300 ml of water. The resulting liquid was placed in another beaker and the two decoctions were mixed. The three disassembled prescriptions were evaporated and concentrated separately.

### 2.2 Experimental animal and cell line

Thirty male BALB/c mice (specific pathogen‐free [SPF], 8 weeks old, body weight 20 ± 2 g) were purchased from Sbeifu Biotechnology Co. Ltd. The animals were freely fed with water and chow under standard conditions. Mouse liver cancer H22 cells were purchased from China Center for Type Culture Collection (CCTCC) (no. 3142C0001000000110). The cells were cultured in a culture medium containing 90% of RPMI‐1640 (GIBCO), 10% of fetal bovine serum (FBS), and 1% of double antibody (GIBCO). Murine H22 cells in the logarithmic growth stage were collected. After centrifugation at 300 ***g***, 4°C, and 5 min, The cells were suspended with RPMI‐1640 basic medium (excluding FBS and double antibodies). The cell concentration was adjusted to ~2 × l0^6^/ml with cell viability of ≥95%.

### 2.3 Establishment of animal model

The H22 tumor‐bearing mice were randomly divided into five groups (*n* = 6 per group), including the control, model, Yangyin prescription (YYP), Fuzheng prescription (FZP), and Jiedu prescription groups (JDP). To establish the HCC mousse model, we subcutaneously injected 2 × 10^6^ H22 cells into the right armpit of BALB/c mice in the three treatment groups and the model group.^18^ After 24 h, intragastric administration of the three disassembled prescriptions or normal saline was performed once a day for 2 weeks. Specifically, YYP (4.5 g/kg body weight), FZP (5.7 g/kg body weight), and JDP (6.2 g/kg body weight) were orally administered to mice in the YYP, FZP, and JDP groups, respectively, in a volume of 0.2 ml. The control and model mice were administered the same volume of saline solution orally.

### 2.4 In vivo antitumor experiment

During treatment, the mice were weighed every 2 days. After the last intragastric administration, the mice were fasted for 12 h and sacrificed by cervical dislocation. The thymus, spleen, and tumors were removed and weighed before washing in normal saline. The peripheral blood of the mice was collected from the retrobulbar venous plexus. The tumor inhibition rate (%) was calculated as follows: tumor inhibition rate = (mean tumor weight of model group—mean tumor weight of treatment group)/mean tumor weight of model group × 100%. Similarly, the thymus or spleen index was calculated by dividing the thymus or spleen weight (g) by the body weight (g).

### 2.5 Hematoxylin and eosin (H&E) staining

The tumors were fixed with 4% paraformaldehyde. After 24 h, the samples were dehydrated with 75% ethanol. The tissues were embedded in paraffin and cut into 4 μm‐thick sections. The sections were dewaxed with xylene, rehydrated with gradient ethanol, and dyed using hematoxylin. Next, the sections were rinsed with running water for 5 min. The sections were immersed in hydrochloric acid alcohol differentiation solution for 30 s, followed by eosin solution for 2 min. The resulting sections were imaged using a light microscope.

### 2.6 Terminal deoxynucleotidyl transferase‐mediated dUTP nick‐end labeling (TUNEL) assay

The tumor tissue sections were deparaffinized with xylene before rehydrating with ethanol via gradient concentration, followed by washing with water. Subsequently, antigen retrieval was performed, specifically, the slides were incubated with proteinase K working solution (stock solution:PBS = 1:9) at 37°C, for 22 min. After washing with PBS (pH 7.4) in a rocker device for 5 min three times, TUNEL staining was performed using the Fluorescein (FITC) TUNEL Cell Apoptosis Detection Kit (Servicebio). Images of the tumor tissue were collected using a fluorescence microscope, and ImageJ 1.53e (NIH) was used to calculate the percentage of TUNEL‐positive cells.

### 2.7 Immunohistochemical staining

The paraffinized sections of tumor tissue were dewaxed with xylene and immersed in gradient ethanol before being rehydrated with water. The tumor tissue was repaired with EDTA antigen retrieval solution (pH 9.0) (Servicebio) in a microwave. After cooling at 25°C, the slides were placed in PBS (pH 7.4) and shaken on a decolorizing shaker for 5 min three times. The slides were immersed in 3% hydrogen peroxide solution for 25 min at RT before washing for 5 min on a decolorizing shaker three times. The slides were incubated with 3% BSA at 25°C for 30 min before incubating with primary antibody, including mouse antibody against CD8 and cleaved caspase‐3. After incubating with secondary antibody for 50 min. The primary antibodies involved in this assay were Cleaved‐caspase3 (Servicebio, GB11532) and CD8 (Cell Signaling Technology, #98941); and the HRP Goat anti‐rabbit secondary antibody was used. DAB chromogenic fluid was used for color development. Images were visualized using fluorescence microscopy.

### 2.8 Mononuclear cells isolation assays

Single‐cell suspensions of lymphocytes from peripheral blood and spleen were obtained. Briefly, samples of peripheral blood were collected from the retrobulbar venous plexus in the mice. Mononuclear cells were isolated using a mouse lymphocyte separation medium (TBD) according to the manufacturer's instructions. The spleens of the H22 tumor‐bearing mice were removed and washed with sterile normal saline (NS). Next, the spleens were mashed using a Teflon pestle and filtered through a sterile 200‐gauge steel mesh. Cell isolation was performed on ice using a precooled solution. The cell suspension was collected into a 15‐ml tube and centrifuged for 5 min at 300 ***g***. After lysing erythrocytes with lysis solution (FACS; BD Biosciences) for 15 min, the cells were washed twice with PBS.

### 2.9 Flow cytometric analysis

Single‐cell suspensions (200 μl, 1 × 10^7^ cells/L) were surface stained with the following anti‐mouse monoclonal antibody in the dark for 30 min at 4°C: BV786‐conjugated anti‐CD3, fluorescein isothiocyanate (FITC)‐conjugated anti‐CD4, BV510‐conjugated anti‐CD8, BV605‐conjugated TIGIT, PE‐conjugated Tim‐3, and APC‐conjugated PD‐1. Flow cytometry analysis was performed using FACSCelesta™ and the data were analyzed using FlowJo (version 10).

### 2.10 Detection of serum cytokines

The level of cytokines in serum was evaluated using a cancer biomarker panel of Milliplex® MAP Mouse High Sensitivity T Cell Magnetic Bead Panel (Millipore) according to the manufacturer's instructions. Peripheral blood was collected from the retrobulbar venous plexus and centrifuged at 800 ***g*** at 4°C for 15 min. The resulting supernatant was collected and 100 μl of each sample was added to a 96‐well plate in triplicate. Next, EMD Millipore beads were coated with the following cytokines: IL‐1β, IL‐2, IL‐13, IL‐10, IL‐4, IL‐6, granulocyte‐macrophage colony‐stimulating factor (GM‐CSF), TNF‐α, and IFN‐γ. The beads were then added to the serum samples and incubated with agitation at 4°C overnight. After incubation, the wells were supplemented with sheath fluid and analyzed using a multiplexing instrument. The fluorescence intensity of the samples was measured by FLEXMAP 3D® System (Luminex) and the density of the cytokines was calculated with a 5‐parameters logistic fitting curve. The production of serum cytokines was measured using Luminex® 200™ Instrument System (Invitrogen) and calculated using a software package of xPONENT (Luminex Corporation).

### 2.11 Establishment of bioactive components database

To further explore the mechanism of the key drugs of YFJP, network pharmacology was used. The traditional Chinese medicine systems pharmacology database and analysis platform (TCMSP) contains 499 TCM items registered in the Chinese Pharmacopeia and 29,384 chemical components. Each ingredient is assigned 12 ADME properties for drug screening and evaluation.^19^ All components of the key drugs were obtained. ADME properties are important indicators of drug absorption, distribution, metabolism, and excretion (ADME). Among these, oral bioavailability (OB) describes the percentage of an oral drug that is absorbed from the gastrointestinal tract and passed through the liver to the systemic circulation.^20^ Similarly, drug‐likeness (DL) is a qualitative concept used to evaluate the likelihood of a compound becoming a drug.^21^ In our current study, OB ≥30% and DL ≥0.18 were considered high OB or DL. The components of key drugs that met these criteria were selected as bioactive components. The chemical structures of each bioactive component were prepared and the Open Babel software (version 2.4.1) was used to convert them into SMILES format.^22^

### 2.12 Identification of drug targets

SwissTargetPrediction was used to predict the targets of bioactive compounds based on the principle of chemical similarity. Specifically, the prediction of the targets of the inputted molecules is based on matching the molecule with proteins with known ligands.^23^

### 2.13 Identification of significant HCC targets

Significant HCC targets were collected from the DisGeNET,^24^ TTD,^25^ Drugbank,^26^ OncoDB.HCC,^27^ and GAD databases,^28^ and the intersection of the five databases was evaluated for further analysis. Venny2.1, an online analysis tool, was used to construct Venn diagrams,^29^ wherein the shared targets of the drugs and HCC were highlighted in the intersection.

### 2.14 Protein–protein interaction data

The STRING database^30^ was used to search for the known and predicted functional partners of the putative targets. Each putative target was input into the STRING database, setting the organism as *Homo sapiens*. For data screening, a confidence score over 0.9 was used.

### 2.15 Network construction

Four networks were established^1^: compound‐target network^2^; protein–protein interaction (PPI) network^3^; gene–gene inner network^4^; compound‐targets‐pathway network. The networks were constructed using the visualization software Cytoscape3.7.2.^31^

### 2.16 Topological analysis of network

Network Analyzer, an internal tool of Cytoscape software, was used to calculate the topological parameters of the networks constructed. The Degree (De) describes the number of nodes that interact with a certain node in the network. The average shortest path length (ASPL) represents the average length of the shortest path between all interacting nodes in the network. The closeness centrality (CC) refers to the reciprocal sum of the shortest distances between a node and other nodes in the network, which depicts the proximity of the node with others. The betweenness centrality (BC) represents the proportion of the shortest paths passing through a node in all shortest paths in the network. The higher the De, CC, and BC values of a node and the lower the ASPL, the more biological functions they participate in and the stronger their biological importance within the network. We retained the highest 20% of the De, CC, and BC, as well as the lowest 20% of the ASPL, for the shared targets for further study.

### 2.17 Clustering analysis of network

The Cytoscape plugins Clustering With Overlapping Neighborhood Expansion (ClusterONE, version 1.0) and Molecular Complex Detection (MCODE, version 1.6.1) were used to identify regions with large weights in the network, which may represent molecular complexes. ClusterONE is used to detect the overlapping nodes in the PPI network.^32^ MCODE is used to detect the densely connected area in the PPI network.^33^ We excavated the highly clustered targets through the plugins, and the shared targets obtained were retained for further study.

### 2.18 Gene ontology (GO) and Kyoto encyclopedia of genes and genomes (KEGG) pathway enrichment analyses

The Cytoscape plugin ClueGo (version 2.5.7) was used for the GO and KEGG pathway enrichment analyses. The putative targets were entered to observe significant KEGG pathways and GO biological process (bp), cellular component (cc), and molecular function (mf) terms. A two‐sided hypergeometric with a *p*‐value ≤0.01 was used as the test type.

### 2.19 Molecular docking

The protein crystal structures of the key targets were obtained from the Protein Data Bank (PDB),^34^ and protein crystal structures with X‐RAY DIFFRACTION <2 were selected. The 3D structure of the ligands was prepared using the PubChem database. AutoDockTool (version 1.5.7) was then used to preprocess the protein crystal structures. In particular, water molecules were deleted and hydrogen ions and charges were added. OpenBable software was used to convert the ligands into .pdb format before preprocessing via AutoDockTools. Next, the active site of the putative proteins was defined. Molecular docking was performed using AutoDock Vina (version 1.2.0). The highest docking score was measured to screen for the optimal combination of components and targets. The highest‐ranking docked pairs were visualized using PyMOL software^35^ (version 2.5.0).

### 2.20 Western blotting

Total protein of tumor tissue from the H22 tumor‐bearing mice was extracted using RIPA lysate containing protease inhibitor and PMSF (Solarbio). Total protein was quantified using the BCA assay. Protein samples were electrophoresed with 10% SDS‐PAGE gel and transferred onto the polyvinylidene fluoride (PVDF) membrane. After blocking with 5% non‐fat milk, the PVDF membranes were incubated with primary antibodies (1:1000) at 4°C overnight. The antibodies used included PI3K, p53, EGFR, and ERK1/2 antibodies. Next, the membranes were incubated with secondary antibodies at room temperature for 1.5 h, followed by washing three times. The immune response area was detected by enhanced chemiluminescence (ECL) (Bio‐Rad) developer. ImageJ software (version 1.53e) (NIH) was used for data analysis.

### 2.21 Statistical analysis

Prism8 software was used for statistical analysis. Variables following normal distribution were analyzed using one‐way ANOVA. Tukey's multiple comparison test was used for post‐hoc testing. Variables following a skewed distribution were compared using Kruskal–Wallis test. A *p*‐value <0.05 was considered statistically significant.

## 3 RESULTS

### 3.1 FZP significantly inhibits tumor growth and restores immune function in vivo

**FIGURE 1:**
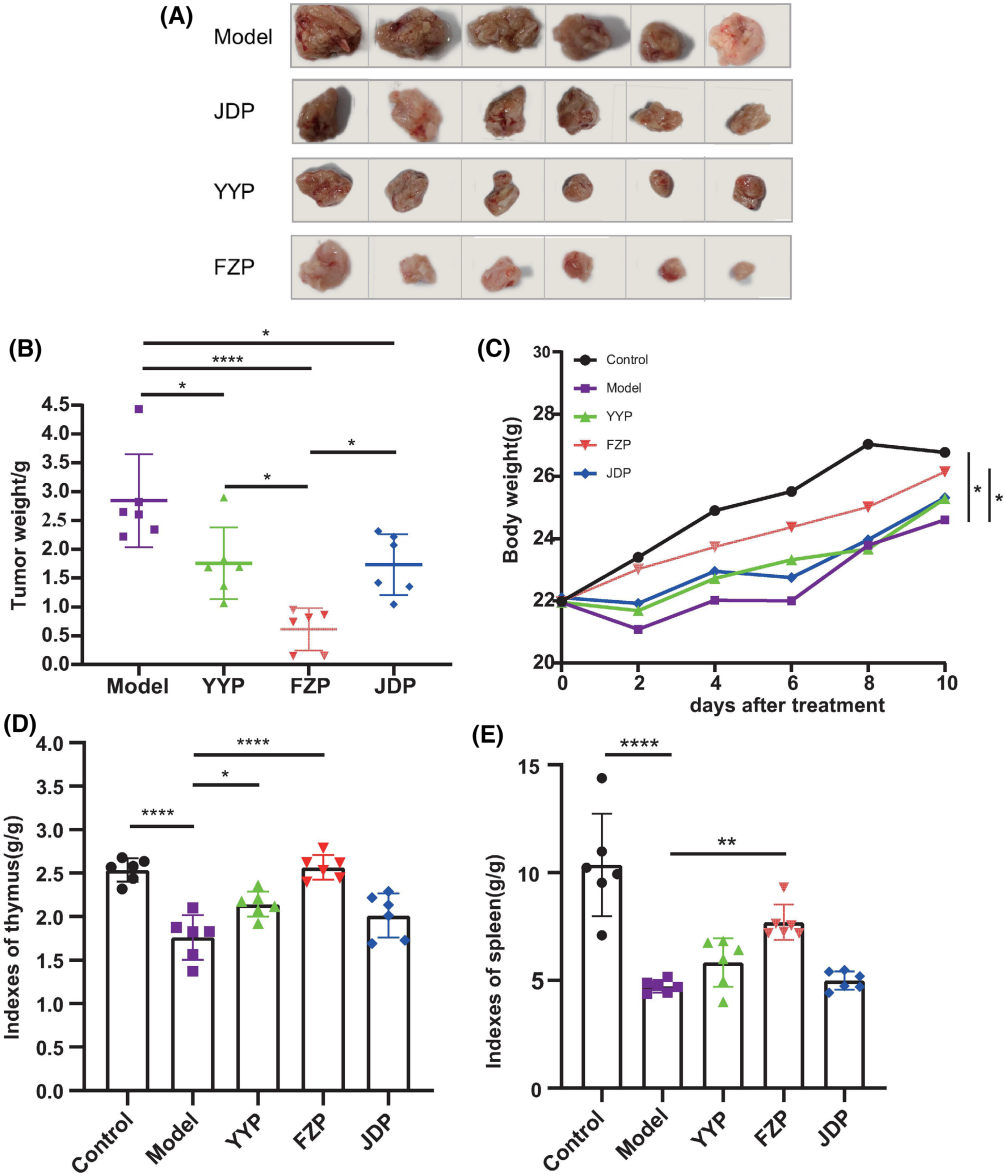
Antitumor effects of the three disassembled prescriptions of YFJP. Thirty H22 tumor‐bearing mice were randomly assigned to the control, model, YYP, FZP, and JDP groups. The tumors, thymus, and spleens of the mice were removed after 2 weeks of oral treatment. Mice were weighed every 2 days during treatment. (A) Tumor appearance was documented by photography. Representative images of each group are shown. (B) Tumor weight among the groups. (C) Change in the body weight of the mice in each group during treatment. (D) Thymus index of the mice in each group. (E) Spleen index of the mice in each group. Data are expressed as the mean ± SD (*n* = 6 for each group): *p < 0.05, **p < 0.01, ****p < 0.0001.

As shown in Figure 1A, tumors in mice in the YYP, FZP, and JDP groups were all reduced, with less red fluid and adhesion compared to that in mice in the model group. In the FZP group, the tumor volume decreased significantly, and part of the tumor capsule was intact. The tumor inhibition rates of the YYP, FZP, and JDP groups were 20.9%, 63.1%, and 25.0%, respectively. Compared with the model group, the tumor weights of the three treatment groups were significantly reduced (*p* < 0.05 or <0.0001). However, tumor weight loss was observed in the JDP and YYP groups (Figure 1C). Interestingly, tumor weight in the FZP group was significantly lower than in the YYP and JDP groups (*p* < 0.05), (Figure 1B,C). Additionally, the thymus index of the model group was significantly decreased compared with the control group (*p* < 0.0001). After treatment, the thymus index of the YYP and FZP groups was significantly increased (*p* < 0.05, <0.0001), while no significant difference was observed between the JDP and model groups (*p* = 0.197) (Figure 1D). The spleen index of the model group was significantly decreased compared with the control group (*p* < 0.0001). After treatment with the three decoctions, the spleen index of the FZP group was significantly increased (*p* < 0.01), while no statistical difference was observed between the JDP or YYP groups and the model group (*p* = 0.996, 0.553) (Figure 1E). These findings indicate that all three disassembled prescriptions of YFJP inhibited tumor growth; however, FZP was the most effective in inhibiting tumor and improving immune function in H22 tumor‐bearing mice.

### 3.2 FZP significantly promotes tumor necrosis and stimulates lymphocyte infiltration

**FIGURE 2:**
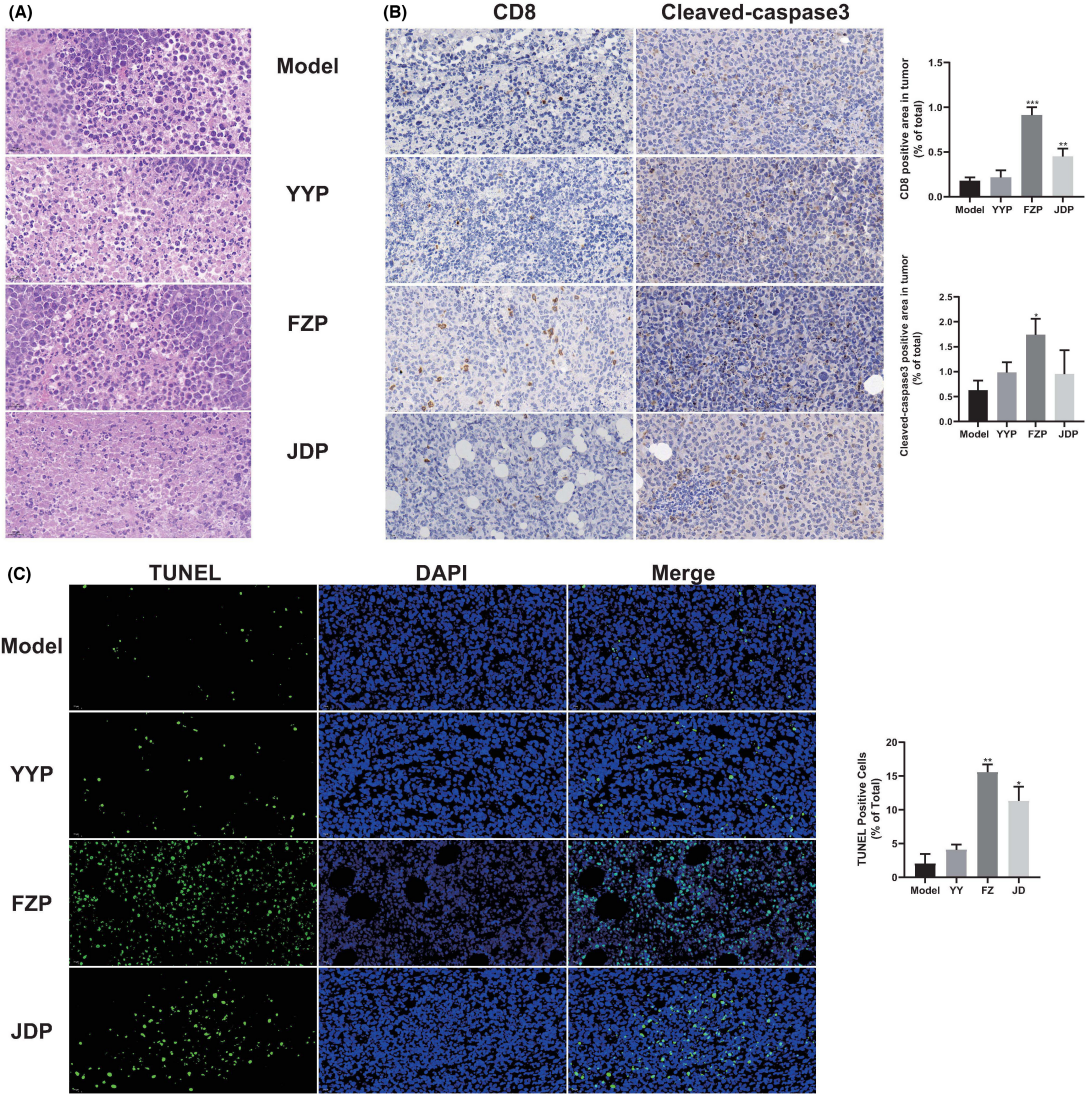
Effects of the three disassembled prescriptions of YFJP on tumor histology and tumor‐infiltrating lymphocytes. (A) The H&E staining of the tumors of the YYP, FZP, and JDP groups was compared with that of the model group (400× magnification). (B) Immunohistochemical staining of CD8 and cleaved caspase‐3 of the tumor tissue of the YYP, FZP, and JDP groups. Scale bar, 50 μm. (C) TUNEL staining of the tumor tissue of the YYP, FZP, and JDP groups. Scale bar, 50 μm. Data are expressed as the mean ± SD: *p < 0.05, **p < 0.01, ***p < 0.001.

To determine the effect of YYP, FZP, and JDP on tumor‐infiltrating lymphocytes (TILs) in H22 tumor‐bearing mice, we observed the excised tumor tissue after H&E staining. As shown in Figure 2A, tumor cells in the model group showed increased volume, significantly increased nuclear‐cytoplasmic ratio, and fewer dead cells. By contrast, more necrotic tissue and various degrees of apoptosis of tumor cells were observed in the three treatment groups. In addition, compared with the model group, different degrees of infiltration of lymphocytes were observed in the three treatment groups, while the number of TILs in the FZP group was increased. The result of immunohistochemistry analysis indicates that the number of CD8^+^ T cells in the FZP group that infiltrated into the solid tumor was significantly increased. Notably, cleaved caspase‐3 staining and the proportion of TUNEL positive cells were also found to be significantly increased in the FZP group (Figure 2B,C), suggesting that FZP promoted the apoptosis of tumor cells. These findings indicate that the antitumor effect of YFJP and the effect of stimulating CD8^+^ T cell infiltration may be based on FZP.

### 3.3 FZP increases the level of T cells in the spleen and peripheral blood of H22 tumor‐bearing mice

**FIGURE 3:**
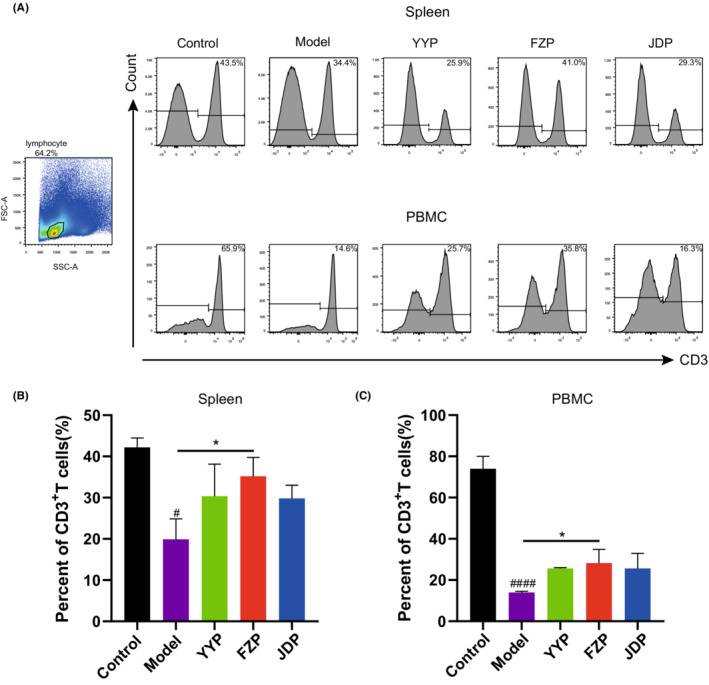
FZP increased the proportion of CD3^+^ T cells in the spleen and peripheral blood of H22 tumor‐bearing mice. (A) Flow cytometry was used to detect the proportion of CD3^+^ T cells in the spleen and peripheral blood. (B) Proportion of CD3^+^ T cells in each group (*n* = 6 in each group): compared with the control group, ^#^ *p* < 0.05, ^####^ *p* < 0.0001; compared with the model group, *p < 0.05.

To evaluate the influence of disassembled prescriptions of YFJP on T cells, the percentage of CD3^+^ T cells in the spleen and peripheral blood was examined after 14 days of treatment using flow cytometry. As shown in Figure 3, compared with the control group, the level of CD3^+^ T cells in the model group decreased in both the peripheral blood and spleen (*p* < 0.0001, <0.05), suggesting that HCC inhibited T cell infiltration in the tumor‐bearing mice model. Compared with the model group, the level of CD3^+^ T cells in the peripheral blood and spleen of the FZP group increased significantly (*p* < 0.05), suggesting that FZP treatment restored lymphocyte infiltration in both organs and blood circulation.

### 3.4 FZP increases the levels of CD8 ^+^ T cells in the peripheral blood and spleen and alleviates the exhaustion phenotype

**FIGURE 4:**
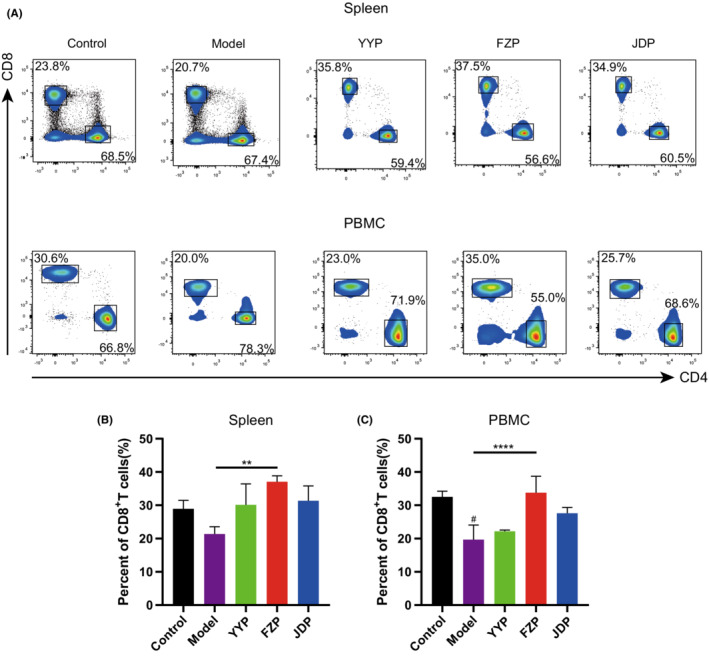
FZP significantly increased the proportion of CD8^+^ T cells in the spleen and peripheral blood of H22 tumor‐bearing mice. (A) Flow cytometry was used to detect the proportion of T cell subsets in the spleen and peripheral blood, including CD4^+^ and CD8^+^ T cells. (B) Proportion of CD8^+^ T cells in the spleen. (C) Proportion of CD8^+^ T cells in the peripheral blood (*n* = 6 in each group): compared with the control group, ^#^ *p* < 0.05; compared with the model group, **p < 0.01, ****p < 0.0001.

**FIGURE 5:**
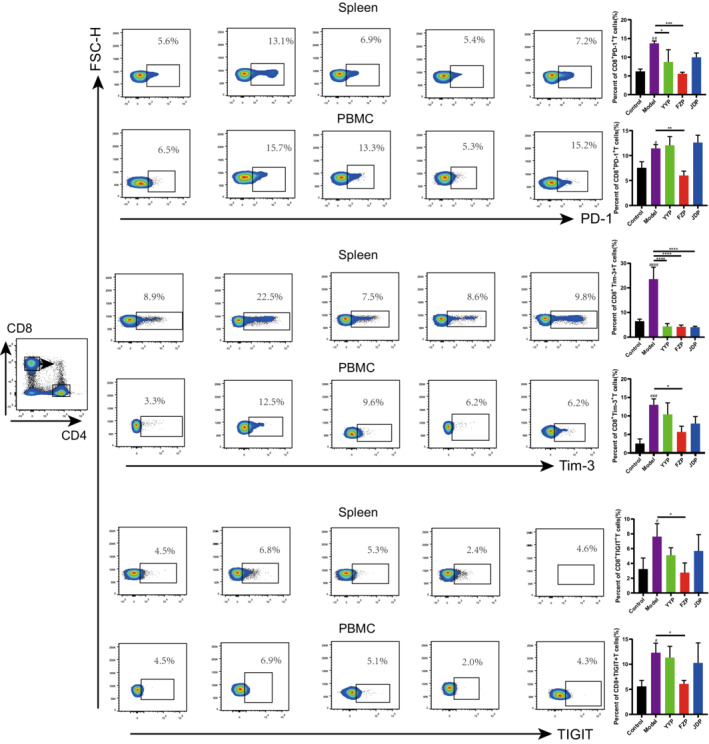
FZP significantly reduced the expression of inhibitory receptors on CD8^+^ T cells in the peripheral blood and spleen of H22 tumor‐bearing mice. Flow cytometry was used to detect the proportion of PD‐1, Tim‐3, and TIGIT in CD8^+^ T cells in each group (*n* = 6 of each group): compared with the control group, ^#^ *p* < 0.05, ^##^ *p* < 0.01, ^###^ *p* < 0.001, ^####^ *p* < 0.0001; compared with the model group, *p < 0.05, **p < 0.01, ***p < 0.001, ****p < 0.0001.

To explore the effects of the disassembled prescriptions on the TME of H22 tumor‐bearing mice, the percentage of CD8^+^ T cells in the spleen and peripheral blood was examined separately. As shown in Figure 4, FZP significantly improved the ratio of CD8^+^ T cells in both the spleen and peripheral blood compared with the model group (*p* < 0.01 or <0.0001). Although CD4^+^ T cells were also detected, the ratio of this subset was not influenced significantly in the spleen or peripheral blood of H22 tumor‐bearing mice. This suggests that FZP treatment achieved an immunoregulatory effect on the HCC microenvironment via the action of CD8^+^ T cells. Next, the expression of inhibitory receptors, including programmed cell death protein 1 (PD‐1), T cell immunoglobulin domain, mucin domain‐3 (Tim‐3), and T cell immunoreceptor with Ig and ITIM domains (TIGIT), on CD8+ T cells in the spleen and peripheral blood was determined using flow cytometry. As shown in Figure 5, compared with the control group, the percentages of CD8^+^PD‐1^+^ T, CD8^+^Tim‐3^+^ T, and CD8^+^TIGIT^+^ T cells were significantly increased in the model group. Compared with the model group, the percentage of CD8^+^PD‐1^+^ T cells decreased in both the spleen and peripheral blood in the FZP group (*p* < 0.001 or *p* < 0.01). Notably, CD8^+^PD‐1+ T cells were also decreased in the YYP group, but only in the spleen (*p* < 0.05). Moreover, YYP, FZP, and JDP all reduced the level of CD8^+^Tim‐3^+^ T cells in the spleen (*p* < 0.0001), but only FZP decreased the expression of Tim‐3 in CD8^+^ T cells in both the spleen and peripheral blood (*p* < 0.05). In addition, the percentage of CD8^+^TIGIT^+^ T cells was significantly reduced in both the spleen and peripheral blood in the FZP group (*p* < 0.05). A representation of the flow gating strategy is provided in Figure S1. These results indicate that the three disassembled prescriptions protected CD8^+^ T cells against exhaustion, among which FZP played the most significant role.

### 3.5 FZP significantly inhibits the production of immunosuppressive cytokines in the peripheral blood

To investigate the regulating effect of the disassembled prescriptions of YFJP on the immunosuppressive HCC microenvironment, the production of pro‐inflammatory, anti‐inflammatory, and effector cytokines of CD8^+^ T cells was detected. The production of IFN‐γ and IL‐2 is a representation of the proliferation and effect of CD8^+^ T cells.^36^ IL‐4, IL‐10, IL‐13, and GM‐CSF are markers of M2 macrophages, which are also anti‐inflammatory cytokines, while IL‐1β, IL‐6, and TNF‐α are markers of M1 macrophages, which are pro‐inflammatory cytokines.^37^ In addition, immunosuppressive cytokines are produced by immunosuppressive cells, such as CAFs or tumor cells. IL‐10 is an important soluble mediator in the TME, responsible for mediating T cell exhaustion.^38^ Moreover, IL‐10 can inhibit IFN‐γ secretion by tumor‐infiltrating CD8^+^ T cells. Blocking IL‐10 has been reported to increase the number and function of cytotoxic CD8^+^ T cells and increase the efficacy of anti‐PD‐1 therapy.^39^, ^40^ In this context, the levels of GM‐CSF, TNF‐α, IFN‐γ, IL‐1β, IL‐2, IL‐4, IL‐6, IL‐10, and IL‐13 in the peripheral blood of H22 tumor‐bearing mice were detected. As shown in Table 1, compared with the control group, the levels of IL‐1β, IL‐4, IL‐6, IL‐10, and IL‐13 in the model group were significantly increased. Meanwhile, the levels of these cytokines in the FZP group were all reduced. Notably, the level of IL‐13 in the YYP group was also decreased compared to the model group. These results indicate that FZP reduced the levels of immunosuppressive and inflammatory cytokines.

**TABLE 1:** Cytokine production in serum of H22 tumor‐bearing mice. Compared with the control group, ^#^ *p* < 0.05; compared with the model group, *p < 0.05

	Control	Model	YY	FZ	JD
GM‐CSF (pg/ml)	3.49 ± 0.40	9.19 ± 4.20	9.21 ± 4.98	3.13 ± 0.13	5.87 ± 1.86
IFN‐γ (pg/ml)	2.08 ± 1.49	0.21 ± 0.04	0.45 ± 0.45	1.15 ± 0.91	0.20 ± 0.13
IL‐1β (pg/ml)	0.79 ± 0.12	1.16 ± 0.19^#^	0.76 ± 0.20	0.55 ± 0.08*	0.87 ± 0.16
IL‐2 (pg/ml)	2.66 ± 1.15	0.76 ± 0.34	0.69 ± 0.07	2.20 ± 0.41	0.47 ± 0.32
IL‐4 (pg/ml)	0.41 ± 0.33	6.46 ± 1.77^#^	1.47 ± 1.32	0.17 ± 0.07*	4.28 ± 3.08
IL‐6 (pg/ml)	3.50 ± 1.27	147.98 ± 50.63^#^	150.47 ± 63.87	24.24 ± 3.74*	64.28 ± 14.21
IL‐10 (pg/ml)	4.70 ± 2.28	86.61 ± 37.63^#^	32.31 ± 22.30	3.20 ± 1.70*	41.51 ± 10.79
IL‐13 (pg/ml)	3.33 ± 1.56	10.63 ± 2.00^#^	5.82 ± 1.60*	4.57 ± 0.80*	6.92 ± 0.28
TNF‐α (pg/ml)	3.17 ± 0.63	0.43 ± 0.33	1.83 ± 0.96	2.21 ± 0.35	0.91 ± 0.62

### 3.6 Compound‐target network and analysis

**FIGURE 6:**
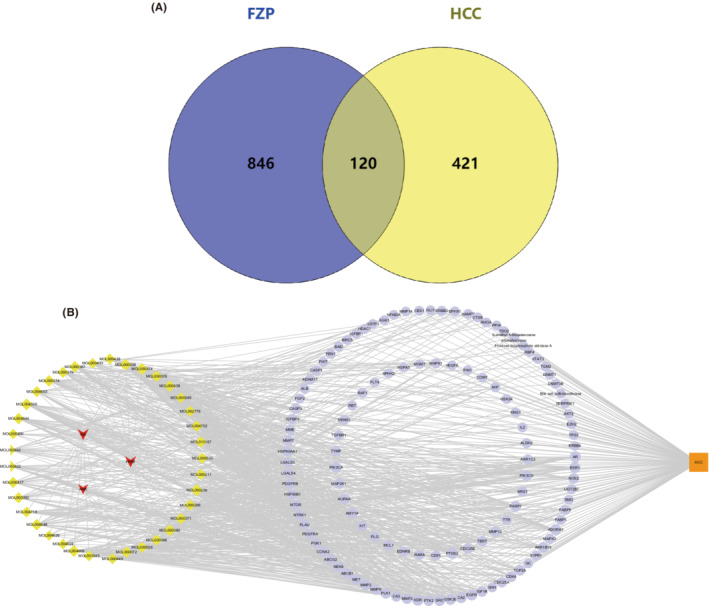
(A) Target mapping for FZP and HCC. (B) Compound‐target network of FZP on HCC. Yellow diamond nodes represent the 37 candidate bioactive ingredients of FZP, blue round nodes represent the 120 putative targets of FZP on HCC, and red V‐shaped nodes represent traditional Chinese medicines contained in FZP. The edges represent the interactions between the nodes.

Considering that FZP showed more significant antitumor and immunoregulation effects in the previous experiment, network pharmacology analysis was used to explore and confirm the mechanism of FZP. Based on the screening criteria (OB ≥30% and DL ≥0.18), we obtained 20 compounds from *Hedysarum multijugum Maxim*. (HMM), 7 compounds from *Atractylodes macrocephala Koidz*. (AMK), and 17 compounds from *Radix Bupleuri* (RB). Details of the drug components are provided in Figure S2. In total, 40 candidate compounds were collected from FZP, and a total of 966 targets of the putative compounds were derived from SwissTargetPrediction. Detailed information on the targets of FZP is provided in Figure S3. Interestingly, quercetin, isorhamnetin, and kaempferol were found to be shared bioactive components of HMM and RB, all three of which are flavonoids. It is well known that flavonoids have a protective effect on HCC, and different flavonoids can play a role in the treatment of HCC by inhibiting tumor growth and exerting anti‐metastasis, anti‐angiogenesis, and anti‐inflammatory effects.^41^ This result is consistent with our previous studies, in which HMM and RB in YFJP were found to improve the suppression of the TME.^42^ To obtain HCC‐related targets, five databases were used. As a result, we obtained 5725 targets from DisGeNet, 35 targets from Drugbank, 40 targets from GAD, 566 targets from onco.DB.HCC, and 42 targets from TTD. Detailed information on these results is provided in Figure S4. The targets shared between the databases were selected, resulting in 541 candidate HCC‐related targets. Then, 120 common targets of FZP and HCC were identified and defined as putative targets (Figure 6A). The putative targets and bioactive compounds were imported into Cytoscape to construct the compound‐target network (Figure 6B). The network consisted of 161 nodes and 878 edges, including HMM, RB, AMK, 37 putative components, and 120 putative targets. Network Analyzer was used to calculate the degree values. The putative compounds were sorted according to the degree value. As a result, jaranol (De = 33), arcapillin (De = 29), 3′,4′,5′,3,5,6,7‐heptamethoxyflavone (De = 29), isorhamnetin (De = 29), kaempferol (De = 28), astrapterocarpan (De = 27), quercetin (De = 27), hederagenin (De = 25), isoflavanone (De = 26), and octalupine (De = 24) were found to be the putative components of FZP in HCC treatment. It is worth noting that all the candidate components belonged to HMM or AMK, suggesting that HMM and AMK in FZP may play a more significant role in the treatment of HCC.

### 3.7 PPI network and analysis

**FIGURE 7:**
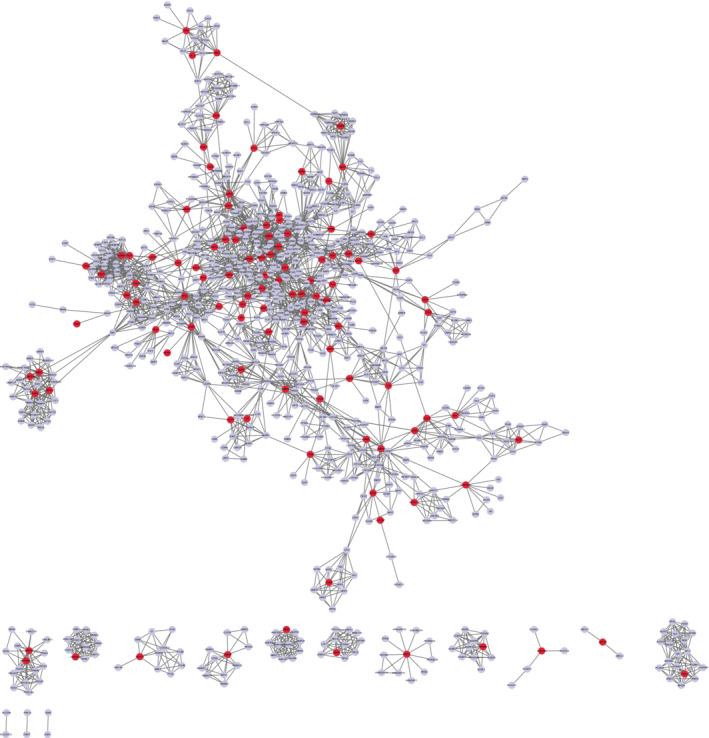
The PPI network for FZP on HCC. Red nodes represent the 120 putative targets of FZP on HCC and blue nodes represent the 652 interactive targets collected in the STRING database of the putative targets. The edges represent the interactions between the nodes.

To explore the hub targets of FZP on HCC, a PPI network was constructed. The 120 putative targets were then imported into the STRING database to identify those associated targets that they directly or indirectly interact with. Based on the screening criteria (combined score of >0.9), a total of 652 interactive targets were derived from the database, and 3845 pairs of interaction relationships were obtained. The resulting targets were used to construct the PPI network, including 652 associated targets and 120 putative targets, as shown in Figure 7. The resulting PPI network consisted of 772 nodes and 3010 edges with an average De of 7.80.

Next, topological and clustering analyses were performed based on the PPI network. To this end, the putative targets were sorted according to the four topological parameters. Then, the top 20% targets for each parameter were combined to obtain a total of 183 topologically important targets. Next, clustering analysis was performed using the Cytoscape plugins MCODE and ClusterONE. Using MCODE, 53 significant clusters and 488 clustering associated targets were obtained. Using ClusterONE, 97 significant clusters and 711 clustering associated targets were obtained. Combining the two sets of targets, a total of 51 targets were filtered. Then, the union of the topologically important targets and the clustering associated targets was observed and a total of 220 targets, including 161 interactive genes and 59 putative targets, were obtained, defined as hub genes.

### 3.8 GO and KEGG analysis

**FIGURE 8:**
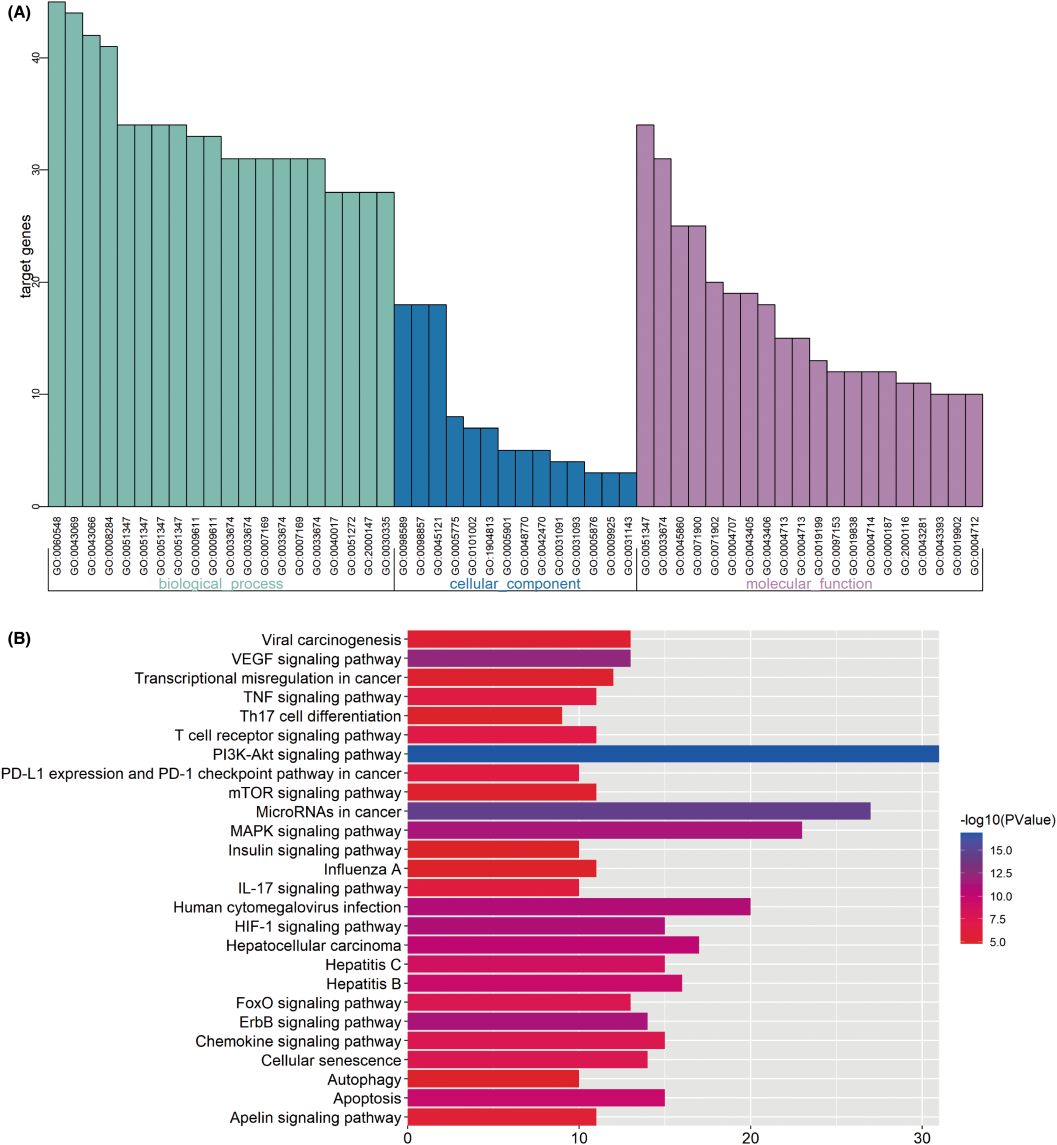
GO and KEGG enrichment analysis based on the putative targets. (A) The Cytoscape plug‐in ClueGO was used to conduct GO enrichment analysis. A total of 715 items of biological process, 14 items of cell composition, and 110 items of molecular function were enriched, and the 20 most significantly enriched items are shown. (B) The Cytoscape plug‐in ClueGO was used to conduct the KEGG analysis. A total of 71 KEGG pathways were enriched, and the most significant 26 HCC‐related pathways are shown.

To further explore the biological mechanisms of the putative targets of FZP in HCC treatment, the Cytoscape plugin ClueGO was used to perform GO analysis. According to the screening criteria of *p* ≤ 0.05, 715 biological process (bp) terms were enriched, including the negative regulation of cell death (GO:0060548), the negative regulation of programmed cell death (GO:0043069), and the negative regulation of apoptotic process (GO:0043066). A total of 14 cellular component (cc) terms were enriched, including the membrane raft (GO:0045121), the membrane microdomain (GO:0098857), and the membrane region (GO:0098589). Finally, 110 molecular function (mf) terms were filtered, including the positive regulation of transferase activity (GO:0051347), the positive regulation of kinase activity (GO:0033674), and the regulation of protein serine/threonine kinase activity (GO:0071900). The terms significantly enriched in three types of GO analysis are shown in Figure 8A. Detailed information on the GO terms is provided in Figure S5. Notably, the effect of FZP on HCC was significantly based on the biological process of the negative regulation of programmed cell death, which is consistent with our previous findings that FZP reduced the overexpression of PD‐1 in CD8^+^ T cells in H22 tumor‐bearing mice.

KEGG pathway analysis based on the 120 putative targets was executed using the same method. As a result, a total of 71 KEGG pathways were enriched (*p* ≤ 0.001), as shown in Figure S5, and a total of 82 putative targets were found to be involved. The most significant HCC‐related signaling pathways are shown in Figure 8B. Notably, PD‐L1 expression and PD‐1 checkpoint pathway in cancer were enriched, consistent with the results of the animal experiments. The relevant putative targets included AKT2, EGFR, MAP2K1, MAPK1, MAPK3, MTOR, NFKBIA, PIK3CA, RAF1, and STAT3. These results suggest that FZP may modulate the PD‐L1/PD‐1 signaling pathway through these ten targets.

### 3.9 Key targets of FZP in HCC treatment

**FIGURE 9:**
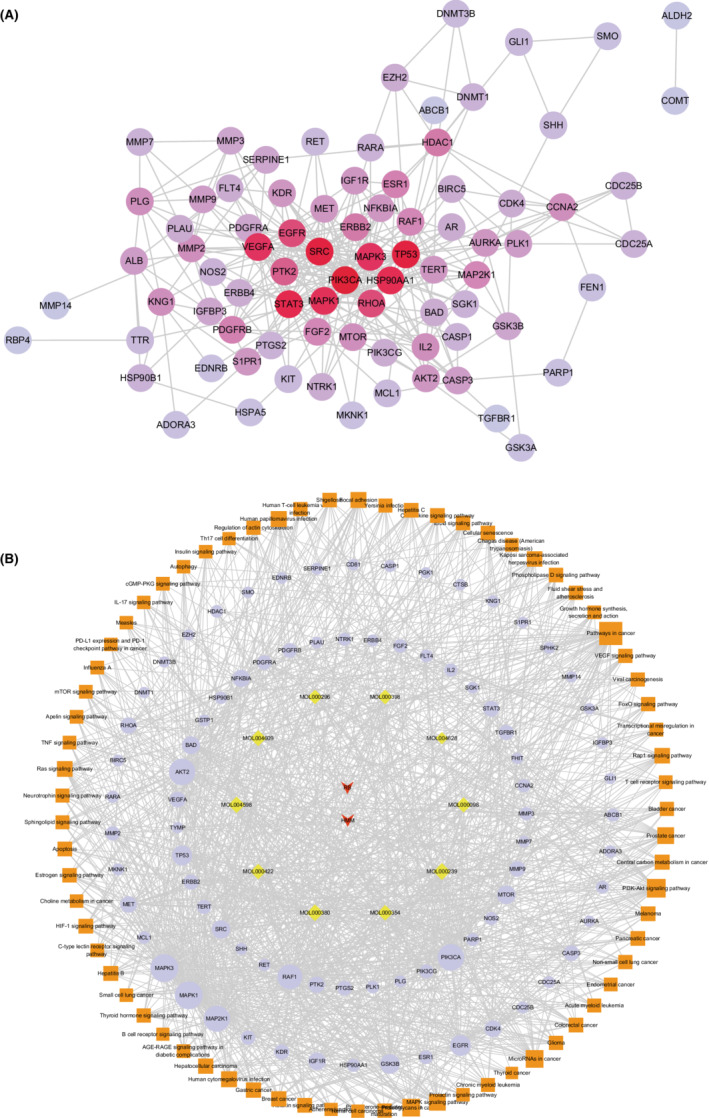
Screening of key targets. (A) The inner network. The network was comprised of 59 hub targets of the PPI network and 82 relevant pathway targets. The color depth of the nodes is proportional to their degree. (B) The component‐target‐pathway network. Orange square nodes represent the 71 KEGG pathways, blue round nodes represent the 120 putative targets of FZP for HCC treatment, yellow diamond nodes represent the 10 putative bioactive ingredients, and the two V‐shaped nodes represent Chinese traditional medicines involved in the putative ingredients and targets of FZP for HCC treatment. Node size is proportional to their degree.

To screen for the key targets, PPI network and KEGG pathway analyses were used to construct a gene–gene inner network (Figure 9A). A total of 82 pathway relevant targets and 59 hub targets were imported into the STRING database to evaluate their interaction relationships. The inner network was constructed using Cytoscape and consisted of 82 nodes and 323 edges. The inner network not only visualized the relationship between the putative targets but also reflected both the results of network analysis and KEGG pathway analysis. Next, topological analysis was used to filter the candidate targets that played the most important role in the inner network, including PIK3CA (De = 30), TP53 (De = 28), SRC (De = 28), STAT3 (De = 26), HSP90AA1 (De = 25), MAPK1 (De = 24), VEGFA (De = 23), MAPK3 (De = 23), EGFR (De = 19), and RHOA (De = 19). Moreover, the 10 candidate components, 82 pathway‐associated targets, and 71 KEGG pathways were imported into Cytoscape to construct a component‐target‐pathway network. As shown in Figure 9B, the network included 165 nodes and 1202 edges. The component‐target‐pathway network indicated that FZP acted on multiple components, multiple targets, and multiple pathways against HCC. The results of the topological analysis indicated that the pathway in cancer had the highest degree value (De = 49). As for the targets, MAPK1 (De = 68), MAPK3 (De = 68), PIK3CA (De = 63), AKT2 (De = 62), MAP2K1 (De = 61), RAF1 (De = 56), TP53 (De = 36), EGFR (De = 40), GSK3B (De = 35), and MTOR (De = 31) were found to play important roles in the network. Lastly, to verify the unbiasedness of the results, the network analysis results of the inner network and the component‐target‐pathway network were combined, the results of which suggested that the five shared targets were the key genes that FZP acted on in the treatment of HCC, namely PIK3CA, TP53, MAPK1, MAPK3, and EGFR.

### 3.10 Western blotting

**FIGURE 10:**
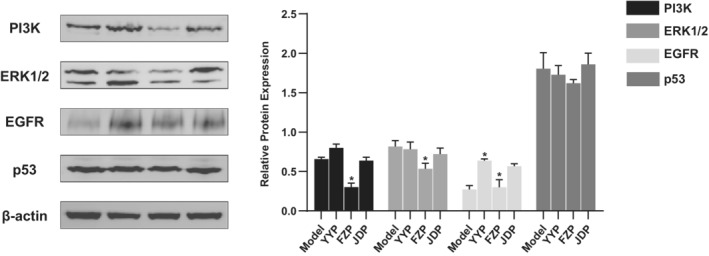
FZP treatment decreased the protein expression of the key targets. H22 tumor‐bearing mice were treated with YYP, FZP, JDP, or NS, and the protein expression of PI3K, EGFR, p53, and ERK1/2 were quantified using western blotting. Data are expressed as the mean ± SD (*n* = 3 per group): *p < 0.05 compared with the model group.

Based on the results of network pharmacology analysis, PIK3CA, EGFR, TP53, MAPK1, and MAPK3 were identified as key targets of FZP in HCC treatment, which was verified using western blotting. As shown in Figure 10, the expression levels of the proteins encoded by the key genes were significantly decreased compared with the model group in the tumor tissue of H22 tumor‐bearing mice (*p* < 0.05). This result suggests that FZP plays a therapeutic role against HCC via the gene targets of PIK3CA, EGFR, TP53, MAPK1, and MAPK3.

### 3.11 Molecular docking verification

**FIGURE 11:**
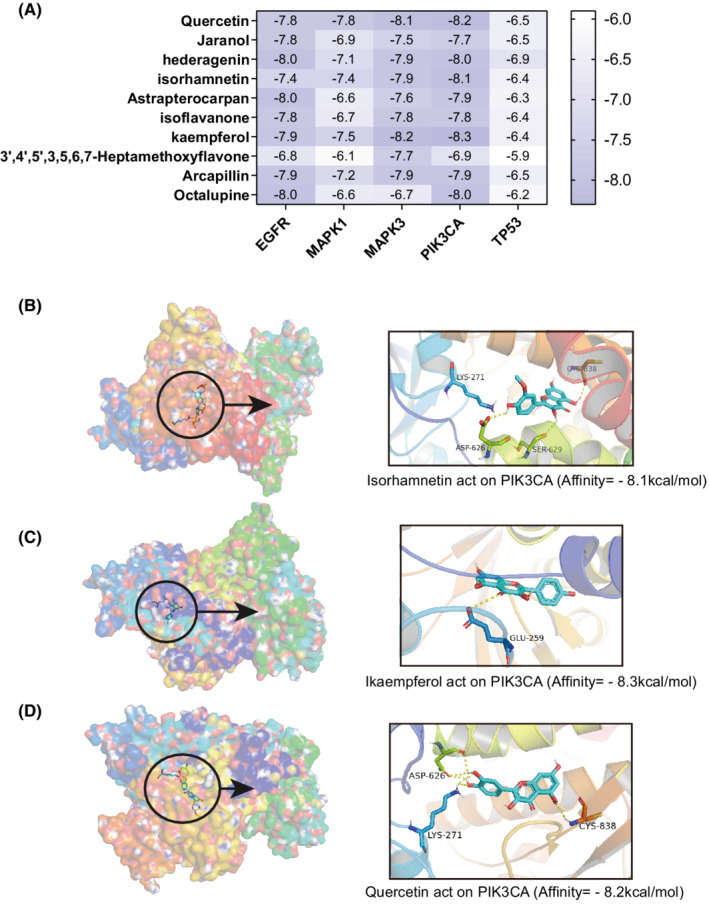
Molecular docking validation of the putative components and key targets. (A) The binding energy of the 50 pairs of molecular docking is shown as a heat map. The color depth of the cell is proportional to the binding activity. (B–D) The three pairs of docking with the strongest binding activity, including isorhamnetin and PIK3CA, kaempferol and PIK3CA, and quercetin and PIK3CA, are shown in 3D.

The docking results of the 10 potential bioactive components and the five key targets are shown in a heat map (Figure 11A), with the corresponding docking scores. The protein crystal structures of IK3CA, TP53, MAPK1, MAPK3, and EGFR were 6oac, 1aie, 6oph, 6ges, and 6v6o, respectively. The lower the docking score, the higher the affinity between the ligand and the target protein. A docking score lower than −5 kcal/mol indicates that the ligand and protein can bind, while a docking score lower than −7 kcal/mol indicates that the binding activity is strong. The results showed that 32 (64%) of the 50 pairs had good binding energy, among which quercetin, kaempferol, and isorhamnetin were the bioactive component with the most strong binding activity. Interestingly, the key target involved in these three pairs of docking results was PIK3CA. Docking was visualized in 3D, as shown in Figure 11B–D. The small molecule compound and the protein target were found to bind in the pocket region of the active site of the protein. The amino acid residues of the target protein were bonded to the active ingredient of the drug via a hydrogen bond. PIK3CA bonded with isorhamnetin via four amino acid residues (CYS‐838, LYS‐271, SER‐629, and ASP‐626), with kaempferol via GLU‐259, and with quercetin via three amino acid residues (ASP‐626, LYS‐271, and CYS‐838).

## 4 DISCUSSION

HCC is believed to develop as a result of interactions between multiple targets and pathways. In this context, the “one drug, one target” strategy often fails to summarize the dynamic balance of the human biological system. TCM is characterized by its multi‐component, multi‐target, and synergistic effects. Potential mechanisms for the treatment of HCC include inducing apoptosis, autophagy, and pyroptosis, as well as the inhibition of tumor cell proliferation, cell cycle arrest, and tumor metabolism regulation.^43^, ^44^ With the recognition of the importance of remodeling the immune microenvironment for the treatment of HCC, the immunomodulatory effects of TCM have increasingly received attention in recent years. Previous studies by our team have shown that YFJP acts on multiple HCC‐related genes to inhibit tumor progression.^42^ In vivo experiments have also confirmed that the mechanism of the antitumor effect of YFJP involves alleviating CD8^+^ T cell exhaustion and improving the immunosuppressive HCC microenvironment.^14^ In the present study, the whole prescription was disassembled into three decoctions to screen the key drugs of YFJP, explore their potential mechanism, and elucidate the therapeutic effects of the disassembled prescriptions on H22 tumor‐bearing mice. Network pharmacology analysis was also used to further investigate the key targets, signaling pathways, and biological processes of the key prescription in the treatment of HCC.

TCM decoctions regulate immunity as a whole, focusing on the complex system of TME and intervening with multiple targets. Several studies have shown that TCM can enhance tumor immunity by stimulating an immune response and removing immunosuppressive factors. The host immune response against cancer is mainly undertaken by T lymphocytes, especially CD8^+^ cytotoxic T lymphocytes. The TCM can upregulate the production of immunostimulatory factors, such as IL‐2, TNF‐α, and IFN‐γ, and reduce the production of immunosuppressive factors, such as IL‐4, L‐10, IL‐13, IL‐6, and TGF‐β, to remodel the TME.^45^ Lu et al. demonstrated that the TCM prescription NHE‐06 did not directly cause tumor cytotoxicity but inhibited the NFκB/IL‐6/STAT3 signaling pathway in vitro and reduced the levels of IL‐6 and IL‐1β in hepa1‐6 tumor‐bearing mice.^46^ The TCM prescription, Shiquanyuzhen decoction has been previously found to increase the thymus and spleen index of H22 tumor‐bearing mice and upregulate the levels of CD8^+^ T cells.^47^ The exhaustion of CD8^+^ T cells is a major challenge in maintaining the antitumor immune response. The most significant feature of exhausted CD8^+^ T cells is a sustained high expression of inhibitory receptors, such as PD‐1, TIGIT, TIM‐3, and LAG3, as well as the progressive loss of effector function.^48^ Our results showed that FZP significantly inhibited tumor growth and promoted tumor cell apoptosis in H22 tumor‐bearing mice, reducing the loss of tumor weight. Among the three disassembled prescriptions of YFJP, FZP stimulated the immune response the most, increasing the immune organ indexes and promoting lymphocyte infiltration in the tumor tissues, peripheral blood, and spleen. Most importantly, FZP showed the greatest inhibition of CD8^+^ T cell exhaustion, downregulating the expression levels of PD‐1, Tim‐3, and TIGIT in CD8^+^ T cells. With regards to reducing the immunosuppressive HCC microenvironment, FZP significantly inhibited the production of immunosuppressive and inflammatory cytokines, including IL‐6, IL‐10, IL‐4, and IL‐13, in the peripheral blood.

The results of the in vivo experiments indicated that YFJP may exert its antitumor and immunoregulatory effects on HCC via FZP. To further explore the potential mechanism, we constructed networks based on FZP and HCC using topological, clustering, and pathway analyses. Five key targets of FZP in HCC treatment were screened and experimentally verified, namely PIK3CA, MAPK1, MAPK3, EGFR, and TP53. The epidermal growth factor receptor (EGFR) is a member of the ERBB family of tyrosine kinase receptors. The EGFR signaling cascade is a key regulator in cell proliferation and cancer development. EGFR is upregulated as a result of mutations in 4% to 66% of patients with HCC and is involved in tumor angiogenesis and proliferation.^49^ Mitogen‐activated protein kinase (MAPK) is a group of serine–threonine protein kinases, among which MAPK1 and MAPK3 act as essential components of the MAP kinase signal transduction pathway. PIK3CA, a component of the PI3K pathway, has been found to play an important role in numerous types of cancers and is considered an oncogene. It is worth noting that the EGFR initiates a signaling cascade through ligand binding and phosphorylation, leading to the activation of the downstream PI3K/AKT and MAPK signaling pathways, which participate in the growth, diffusion, and survival of cells under physiological conditions. The dysregulation of the PI3K/AKT and MAPK signaling pathways is associated with hepatocyte tumorigenesis^[4,49]^. Tumor protein P53 plays a role in tumor suppression in many types of tumors, which can induce apoptosis and growth arrest in different physiological environments. The TP53 gene is the most commonly mutated in patients with HCC, with TP53 mutations dysregulating the cell cycle. Generally, HCC can be classified into the CTNNB1 and TP53 phenotypes, wherein the latter is often characterized by poor differentiation, vascular invasion, multinucleation, and pleomorphism.^50^, ^51^

Combining the results of the GO and KEGG enrichment analyses with the results of the in vivo experiments indicates that FZP exerts its antitumor effect through PD‐L1 expression and the PD‐1 checkpoint pathway in cancer, as well as the biological process of negative regulation of cell death or programmed cell death. The inhibition of the PD‐1/PD‐L1 pathway is a core regulatory mechanism of CD8^+^ T cell exhaustion.^52^, ^53^ The exhaustion of T cells occurs in chronic infection and cancer, where continued antigen exposure leads to the sustained overexpression of PD‐1 in CD8^+^ T cells.

The results of network analysis and molecular docking confirmed that quercetin, kaempferol, and isorhamnetin were the key bioactive components of FZP in the treatment of HCC. These three compounds are flavonoids and shared components of HMM and RB. Studies have shown that quercetin inhibits hepatoma cell growth via p53‐dependent cell cycle arrest and apoptosis. In addition, quercetin produces sustained inhibition of HepG2 extracellular regulatory kinase and proliferation‐related protein Akt.^45^, ^54^ In vitro experiments have shown that quercetin may partially inhibit AKT/mTOR pathway and activate the MAPK pathway, thus stimulating autophagy and inducing hepatocellular carcinoma cell apoptosis.^55^ Isorhamnetin is a metabolite of quercetin that can inhibit liver cancer cells. Studies have shown that isorhamnetin can reduce the phosphorylation of cell proliferation‐related proteins AKT and ERK, increase the expression of apoptosis‐related proteins, such as Bax, induce apoptosis, and inhibit the proliferation of HepG2 cells in vitro by blocking the cells in G_0_‐G_1_ phase.^56^ In addition, isorhamnetin exerts hepatoprotective activity and can inhibit apoptosis and autophagy by regulating the downstream protein P38 of the MAPK pathway, playing a protective role in the liver.^57^ In vivo studies have confirmed that isorhamnetin can inhibit the formation of liver fibrosis, an important risk factor for liver cancer.^58^ Similarly, kaempferol is a major flavonoid aglycone that exerts a variety of antitumor mechanisms.^59^ Many studies have shown that kaempferol can inhibit the proliferation of Huh7 and SK‐HEP‐1 cells, as well as induce hepatoma cell apoptosis and cell cycle arrest.^60^

This study has some potential limitations. Firstly, there was a limited number of mice in each group of the H22 tumor‐bearing model, which may lead to bias in evaluating the drug resistance and survival of mice to TCM decoction. Secondly, this study is based on the results of in vivo experiments for further network pharmacological analysis. Therefore, the mechanism elucidated in this study needs to be verified using in vitro models and human samples in future studies.

## 5 CONCLUSION

In this study, FZP was identified as the key drug in YFJP to relieve T cell exhaustion and improve the immunosuppressive microenvironment, with quercetin, kaempferol, and isorhamnetin as the key bioactive components. FZP regulates HCC‐related signaling pathways, including the PIK3‐Akt signaling pathway, PD‐L1 expression, and PD‐1 checkpoint pathway in cancer, to treat HCC. The therapeutic targets of FZP include PIK3CA, TP53, MAPK1, MAPK3, and EGFR, and FZP positively regulates the molecular functions of transferases and kinases on the cell surface through the membrane raft, membrane microarea, and other cell components to realize the inhibition of cell death and programmed cell death. These results provide evidence for the role of TCM in immunoregulation and the remodeling of the immunosuppressive microenvironment for the treatment of cancer.

## AUTHOR CONTRIBUTIONS

Zhiyun Yang and Weihong Li designed the study. Yuqing Xie, Fengna Yan, and Xinhui Wang performed the animal experiment and the network pharmacological data analysis, as well as prepared the initial draft of the manuscript. Lihua Yu, Huiwen Yan, and Qing Pu collated and constructed our network pharmacological database. All authors have read and approved the final submitted manuscript. All data were generated in‐house, and no paper mill was used. All authors agree to be accountable for all aspects of work ensuring integrity and accuracy.

## CONFLICT OF INTEREST

The authors declare that they have no conflict of interest.

## ETHICAL STATEMENT

The use of experimental animals was in conformance with the Animal Ethics Committee of the Animal Care and Use Committee of Beijing University of Chinese Medicine and strictly operated according to the Guide for the Care and Use of Laboratory Animals published by the US National Institutes of Health (license no. BUCM‐4‐2,021,062,301‐2067).

## Supporting information


Appendix S1

Appendix S2

Appendix S3

Appendix S4

Appendix S5

Appendix S6

## Data Availability

TTD http://db.idrblab.net/ttd/, Drugbank https://www.drugbank.ca/, DisGeNET https://www.disgenet.org/, GAD https://geneticassociationdb.nih.gov/, OncoDB.HCC http://oncodb.hcc.ibms.sinica.edu.tw/index.htm, Liverome http://liverome.kobic.re.kr/index.php, Uniprot http://www.uniprot.org/, STRING https://string‐db.org/, TCMSP http://tcmspw.com/tcmsp.php, PubChem https://pubchem.ncbi.nlm.nih.gov/, Openbabel http://openbabel.org/wiki/Main_Page, SwissTargetPrediction http://www.swisstargetprediction.ch/, PharmMapper http://lilab‐ecust.cn/pharmmapper/, ChemMapper http://lilab‐ecust.cn/chemmapper/, Cytoscape http://www.cytoscape.org/, PDB(protein data bank) http://www.rcsb.org/pdb/home/home.do, AutoDock MGL http://mgltools.scripps.edu/, AutoDock Vina http://vina.scripps.edu/index.html, PyMOL https://pymol.org/2/Abstract.

## References

[1] Siegel RL , Miller KD , Fuchs HE , Jemal A . Cancer statistics, 2021 [published correction appears in CA cancer J clin. 2021 Jul;71(4):359]. CA Cancer J Clin 2021;71(1):7–33. doi:10.3322/caac.21654 33433946

[2] Petrick JL , Florio AA , Znaor A , et al. International trends in hepatocellular carcinoma incidence, 1978–2012. Int J Cancer. 2020;147(2):317‐330. doi:10.1002/ijc.32723 31597196 PMC7470451

[3] Borgia M , Dal Bo M , Toffoli G . Role of virus‐related chronic inflammation and mechanisms of cancer immune‐suppression in pathogenesis and progression of hepatocellular carcinoma. Cancers (Basel). 2021;13(17):4387. doi:10.3390/cancers13174387 34503196 PMC8431318

[4] Llovet JM , Kelley RK , Villanueva A , et al. Hepatocellular carcinoma. Nat Rev Dis Primers. 2021;7(1):6. doi:10.1038/s41572-020-00240-3 33479224 PMC13537433

[5] Villanueva A . Hepatocellular carcinoma. N Engl J Med. 2019;380(15):1450‐1462. doi:10.1056/NEJMra1713263 30970190

[6] Zhang Z , Liu S , Zhang B , Qiao L , Zhang Y , Zhang Y . T cell dysfunction and exhaustion in cancer. Front Cell Dev Biol. 2020;8:17. doi:10.3389/fcell.2020.00017 32117960 PMC7027373

[7] Reeves E , James E . Antigen processing and immune regulation in the response to tumours. Immunology. 2017;150(1):16‐24. doi:10.1111/imm.12675 27658710 PMC5341504

[8] Roderburg C , Özdirik B , Wree A , Demir M , Tacke F . Systemic treatment of hepatocellular carcinoma: from sorafenib to combination therapies Hepat Oncol 2020;7(2):HEP20. doi:10.2217/hep-2020-0004 PMC7338920 32647565

[9] Yu X , Li W , Young KH , Li Y . Posttranslational modifications in PD‐L1 turnover and function: from cradle to grave. Biomedicines. 2021;9(11):1702. doi:10.3390/biomedicines9111702 34829931 PMC8615371

[10] Wang X , Guo G , Guan H , Yu Y , Lu J , Yu J . Challenges and potential of PD‐1/PD‐L1 checkpoint blockade immunotherapy for glioblastoma. J Exp Clin Cancer Res. 2019;38(1):87. doi:10.1186/s13046-019-1085-3 30777100 PMC6380009

[11] Sun L , Fahey P , Zhu X , et al. A cohort study to examine the use of Chinese herbal medicine in combination with conventional therapies for patients with hepatocellular carcinoma in China. Integr Cancer Ther. 2018;17(3):902‐911. doi:10.1177/1534735418775819 29775121 PMC6142107

[12] Lu HL , Su YC , Lin MC , Sun MF , Huang ST . Integrating Chinese and Western medicines reduced the incidence of hepatocellular carcinoma in patients with diabetes mellitus: a Taiwanese population‐based cohort study. Complement Ther Med. 2020;49(102):332. doi:10.1016/j.ctim.2020.102332 32147062

[13] Liu X , Li M , Wang X , et al. Effects of adjuvant traditional Chinese medicine therapy on long‐term survival in patients with hepatocellular carcinoma. Phytomedicine. 2019;62:152930. doi:10.1016/j.phymed.2019.152930 31128485

[14] Yan F , Wang X , Xie Y , et al. Yangyin Fuzheng Jiedu prescription exerts anti‐tumor immunity in hepatocellular carcinoma by alleviating exhausted T cells. Phytomedicine. 2021;91:153722. doi:10.1016/j.phymed.2021.153722 34488188

[15] Yuan H , Ma Q , Cui H , et al. How can synergism of traditional medicines benefit from network pharmacology? Molecules. 2017;22(7):1135. doi:10.3390/molecules22071135 28686181 PMC6152294

[16] Hopkins AL . Network pharmacology. Nat Biotechnol. 2007;25(10):1110‐1111. doi:10.1038/nbt1007-1110 17921993

[17] Boezio B , Audouze K , Ducrot P , Taboureau O . Network‐based approaches in pharmacology. Mol Inform. 2017;36:10. doi:10.1002/minf.201700048 28692140

[18] Li W , Xu Q , He YF , et al. Anti‐tumor effect of steamed codonopsis lanceolata in H22 tumor‐bearing mice and its possible mechanism. Nutrients. 2015;7(10):8294‐8307. doi:10.3390/nu7105395 26426041 PMC4632415

[19] Ru J , Li P , Wang J , et al. TCMSP: a database of systems pharmacology for drug discovery from herbal medicines. J Cheminform. 2014;6:13. doi:10.1186/1758-2946-6-13 24735618 PMC4001360

[20] An L , Lin Y , Li L , et al. Integrating network pharmacology and experimental validation to investigate the effects and mechanism of astragalus flavonoids against hepatic fibrosis. Front Pharmacol. 2021;11:618262. doi:10.3389/fphar.2020.618262 33551818 PMC7862122

[21] Zali H , Rezaei Tavirani M . Meningioma protein–protein interaction network. Arch Iran Med. 2014;17(4):262‐272. 24724603

[22] O'Boyle NM , Banck M , James CA , Morley C , Vandermeersch T , Hutchison GR . Open Babel: an open chemical toolbox. J Cheminform. 2011;3:33. doi:10.1186/1758-2946-3-33 21982300 PMC3198950

[23] Gfeller D , Grosdidier A , Wirth M , Daina A , Michielin O , Zoete V . SwissTargetPrediction: a web server for target prediction of bioactive small molecules. Nucleic Acids Res. 2014;42(Web Server issue):W32‐W38. doi:10.1093/nar/gku293 24792161 PMC4086140

[24] Piñero J , Bravo À , Queralt‐Rosinach N , et al. DisGeNET: a comprehensive platform integrating information on human disease‐associated genes and variants. Nucleic Acids Res. 2017;45(D1):D833‐D839. doi:10.1093/nar/gkw943 27924018 PMC5210640

[25] Chen X , Ji ZL , Chen YZ . TTD: therapeutic target database. Nucleic Acids Res. 2002;30(1):412‐415. doi:10.1093/nar/30.1.412 11752352 PMC99057

[26] Wishart DS , Knox C , Guo AC , et al. DrugBank: a knowledgebase for drugs, drug actions and drug targets. Nucleic Acids Res. 2008;36(Database issue):D901‐D906. doi:10.1093/nar/gkm958 18048412 PMC2238889

[27] Lee L , Wang K , Li G , et al. Liverome: a curated database of liver cancer‐related gene signatures with self‐contained context information. BMC Genomics. 2011;12(Suppl 3):S3. doi:10.1186/1471-2164-12-S3-S3 PMC3333186 22369201

[28] Yasser N , Karam A . GAD: a python script for dividing genome annotation files into feature‐based files. Interdiscip Sci. 2020;12(3):377‐381. doi:10.1007/s12539-020-00378-4 32524530

[29] Wang F , Wang L , Liu F , et al. Investigation of the mechanism of the reduction of anthracycline‐induced cardiotoxicity by Qishen Huanwu capsule based on network pharmacology. Ann Palliat Med. 2021;10(1):16‐28. doi:10.21037/apm-20-2204 33474949

[30] Szklarczyk D , Gable AL , Lyon D , et al. STRING v11: protein–protein association networks with increased coverage, supporting functional discovery in genome‐wide experimental datasets. Nucleic Acids Res. 2019;47(D1):D607‐D613. doi:10.1093/nar/gky1131 30476243 PMC6323986

[31] Shannon P , Markiel A , Ozier O , et al. Cytoscape: a software environment for integrated models of biomolecular interaction networks. Genome Res. 2003;13(11):2498‐2504. doi:10.1101/gr.1239303 14597658 PMC403769

[32] Nepusz T , Yu H , Paccanaro A . Detecting overlapping protein complexes in protein–protein interaction networks. Nat Methods. 2012;9(5):471‐472. doi:10.1038/nmeth.1938 22426491 PMC3543700

[33] Bader GD , Hogue CW . An automated method for finding molecular complexes in large protein interaction networks. BMC Bioinformatics. 2003;4:2. doi:10.1186/1471-2105-4-2 12525261 PMC149346

[34] Burley SK , Berman HM , Kleywegt GJ , Markley JL , Nakamura H , Velankar S . Protein data Bank (PDB): the single global macromolecular structure archive. Methods Mol Biol. 2017;1607:627‐641. doi:10.1007/978-1-4939-7000-1_26 28573592 PMC5823500

[35] Seeliger D , de Groot BL . Ligand docking and binding site analysis with PyMOL and Autodock/Vina. J Comput Aided Mol Des. 2010;24(5):417‐422. doi:10.1007/s10822-010-9352-6 20401516 PMC2881210

[36] Sa Q , Woodward J , Suzuki Y . IL‐2 produced by CD8+ immune T cells can augment their IFN‐γ production independently from their proliferation in the secondary response to an intracellular pathogen. J Immunol. 2013;190(5):2199‐2207. doi:10.4049/jimmunol.1202256 23359502 PMC3578122

[37] Liu S , Zhang C , Maimela NR , et al. Molecular and clinical characterization of CD163 expression via large‐scale analysis in glioma. Oncoimmunology. 2019;8(7):1601478. doi:10.1080/2162402X.2019.1601478 31143523 PMC6527268

[38] Chen J , Ye Y , Liu P , et al. Suppression of T cells by myeloid‐derived suppressor cells in cancer. Hum Immunol. 2017;78(2):113‐119. doi:10.1016/j.humimm.2016.12.001 27939507

[39] Brooks DG , Ha SJ , Elsaesser H , Sharpe AH , Freeman GJ , Oldstone MB . IL‐10 and PD‐L1 operate through distinct pathways to suppress T‐cell activity during persistent viral infection. Proc Natl Acad Sci USA. 2008;105(51):20428‐20433. doi:10.1073/pnas.0811139106 19075244 PMC2629263

[40] Li L , Ma Y , Xu Y . Follicular regulatory T cells infiltrated the ovarian carcinoma and resulted in CD8 T cell dysfunction dependent on IL‐10 pathway. Int Immunopharmacol. 2019;68:81‐87. doi:10.1016/j.intimp.2018.12.051 30616170

[41] García ER , Gutierrez EA , de Melo FCSA , Novaes RD , Gonçalves RV . Flavonoids effects on hepatocellular carcinoma in murine models: a systematic review. Evid Based Complement Alternat Med. 2018;2018:6328970. doi:10.1155/2018/6328970 29681978 PMC5850900

[42] Yan F , Feng M , Wang X , et al. Molecular targets of Yangyin Fuzheng Jiedu prescription in the treatment of hepatocellular carcinoma based on network pharmacology analysis. Cancer Cell Int. 2020;20(1):540. doi:10.1186/s12935-020-01596-y 33292207 PMC7650191

[43] Santhakumar C , Gane EJ , Liu K , McCaughan GW . Current perspectives on the tumor microenvironment in hepatocellular carcinoma. Hepatol Int. 2020;14(6):947‐957. doi:10.1007/s12072-020-10,104-3 33188512

[44] Jia W , Wang L . Using traditional Chinese medicine to treat hepatocellular carcinoma by targeting tumor immunity. Evid Based Complement Alternat Med. 2020;2020:9843486. doi:10.1155/2020/9843486 32595757 PMC7305542

[45] Hu Y , Wang S , Wu X , et al. Chinese herbal medicine‐derived compounds for cancer therapy: a focus on hepatocellular carcinoma. J Ethnopharmacol. 2013;149(3):601‐612. doi:10.1016/j.jep.2013.07.030 23916858

[46] Lu X , Wo G , Li B , et al. The anti‐inflammatory NHE‐06 restores antitumor immunity by targeting NF‐κB/IL‐6/STAT3 signaling in hepatocellular carcinoma. Biomed Pharmacother. 2018;102:420‐427. doi:10.1016/j.biopha.2018.03.099 29574282

[47] Zhu YC , Zhan GJ , Yuan DP , Yang FM , Huang Q . Effects of Shiquanyuzhentang on immunologic function of H_22_ tumor‐bearing mouse. Zhongguo Ying Yong Sheng Li Xue Za Zhi 2017;33(1):51–55. doi:10.12047/j.cjap.5448.2017.012 29926607

[48] Makaremi S , Asadzadeh Z , Hemmat N , et al. Immune checkpoint inhibitors in colorectal cancer: challenges and future prospects. Biomedicines. 2021;9(9):1075. doi:10.3390/biomedicines9091075 34572263 PMC8467932

[49] Couri T , Pillai A . Goals and targets for personalized therapy for HCC. Hepatol Int. 2019;13(2):125‐137. doi:10.1007/s12072-018-9919-1 30600478

[50] Chiang DY , Villanueva A , Hoshida Y , et al. Focal gains of VEGFA and molecular classification of hepatocellular carcinoma. Cancer Res. 2008;68(16):6779‐6788. doi:10.1158/0008-5472.CAN-08-0742 18701503 PMC2587454

[51] Calderaro J , Couchy G , Imbeaud S , et al. Histological subtypes of hepatocellular carcinoma are related to gene mutations and molecular tumour classification. J Hepatol. 2017;67(4):727‐738. doi:10.1016/j.jhep.2017.05.014 28532995

[52] Pauken KE , Wherry EJ . Overcoming T cell exhaustion in infection and cancer. Trends Immunol. 2015;36(4):265‐276. doi:10.1016/j.it.2015.02.008 25797516 PMC4393798

[53] Relecom A , Merhi M , Inchakalody V , et al. Emerging dynamics pathways of response and resistance to PD‐1 and CTLA‐4 blockade: tackling uncertainty by confronting complexity. J Exp Clin Cancer Res. 2021;40(1):74. doi:10.1186/s13046-021-01872-3 33602280 PMC7893879

[54] Ji Y , Li L , Ma YX , et al. Quercetin inhibits growth of hepatocellular carcinoma by apoptosis induction in part via autophagy stimulation in mice. J Nutr Biochem. 2019;69:108‐119. doi:10.1016/j.jnutbio.2019.03.018 31078904 PMC9659433

[55] Wu L , Li J , Liu T , et al. Quercetin shows anti‐tumor effect in hepatocellular carcinoma LM3 cells by abrogating JAK2/STAT3 signaling pathway. Cancer Med. 2019;8(10):4806‐4820. doi:10.1002/cam4.2388 31273958 PMC6712453

[56] Gong G , Guan YY , Zhang ZL , et al. Isorhamnetin: a review of pharmacological effects. Biomed Pharmacother. 2020;128:110301. doi:10.1016/j.biopha.2020.110301 32502837

[57] Lu X , Liu T , Chen K , et al. Isorhamnetin: a hepatoprotective flavonoid inhibits apoptosis and autophagy via P38/PPAR‐α pathway in mice. Biomed Pharmacother. 2018;103:800‐811. doi:10.1016/j.biopha.2018.04.016 29684859

[58] Yang JH , Kim SC , Kim KM , et al. Isorhamnetin attenuates liver fibrosis by inhibiting TGF‐β/Smad signaling and relieving oxidative stress. Eur J Pharmacol. 2016;783:92‐102. doi:10.1016/j.ejphar.2016.04.042 27151496

[59] Imran M , Salehi B , Sharifi‐Rad J , et al. Kaempferol: a key emphasis to its anticancer potential. Molecules. 2019;24(12):2277. doi:10.3390/molecules24122277 31248102 PMC6631472

[60] Mylonis I , Lakka A , Tsakalof A , Simos G . The dietary flavonoid kaempferol effectively inhibits HIF‐1 activity and hepatoma cancer cell viability under hypoxic conditions. Biochem Biophys Res Commun. 2010;398(1):74‐78. doi:10.1016/j.bbrc.2010.06.038 20558139

