# Supplementary figures and images for: Mechanisms and network pharmacological analysis of Yangyin Fuzheng Jiedu prescription in the treatment of hepatocellular carcinoma
Source: Cancer Med. 2022 Aug 31;12(3):3237–59. doi: 10.1002/cam4.5064 (PMC9939140; doi:10.1002/cam4.5064)

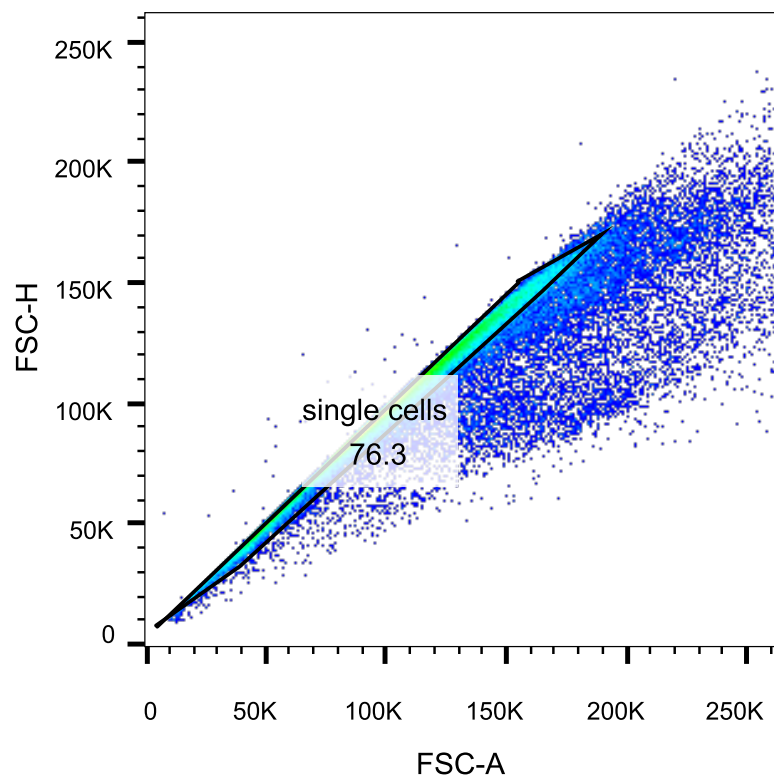

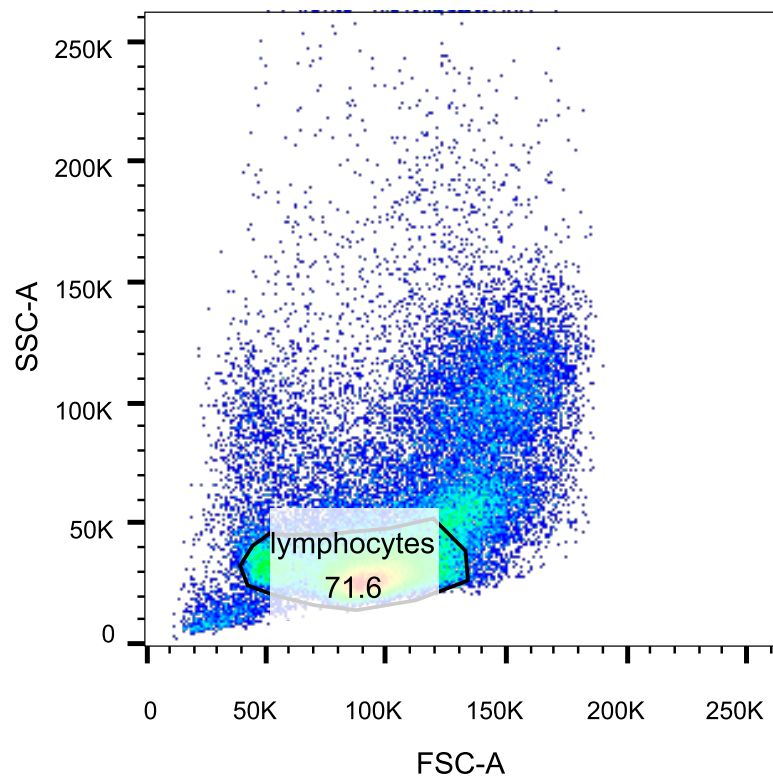

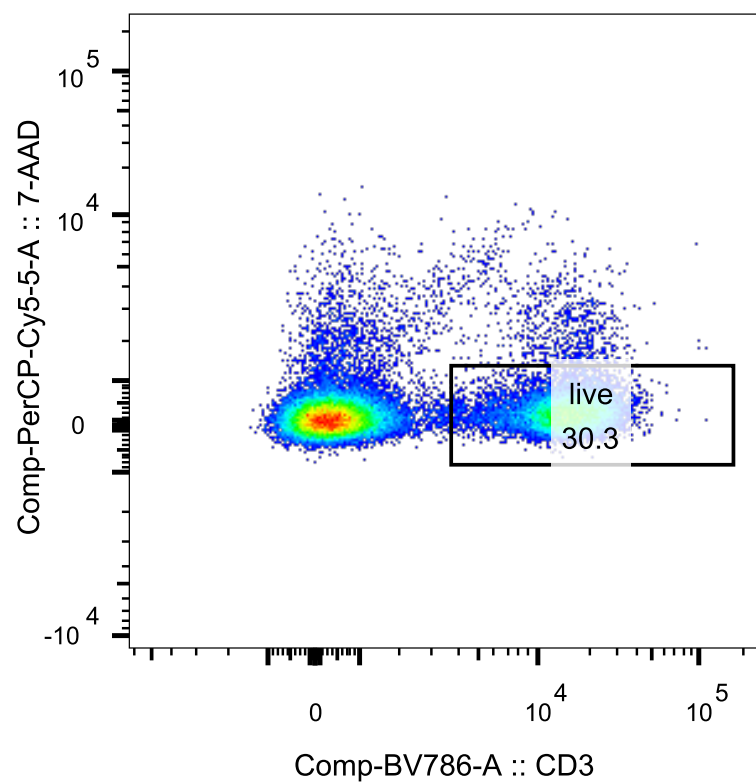

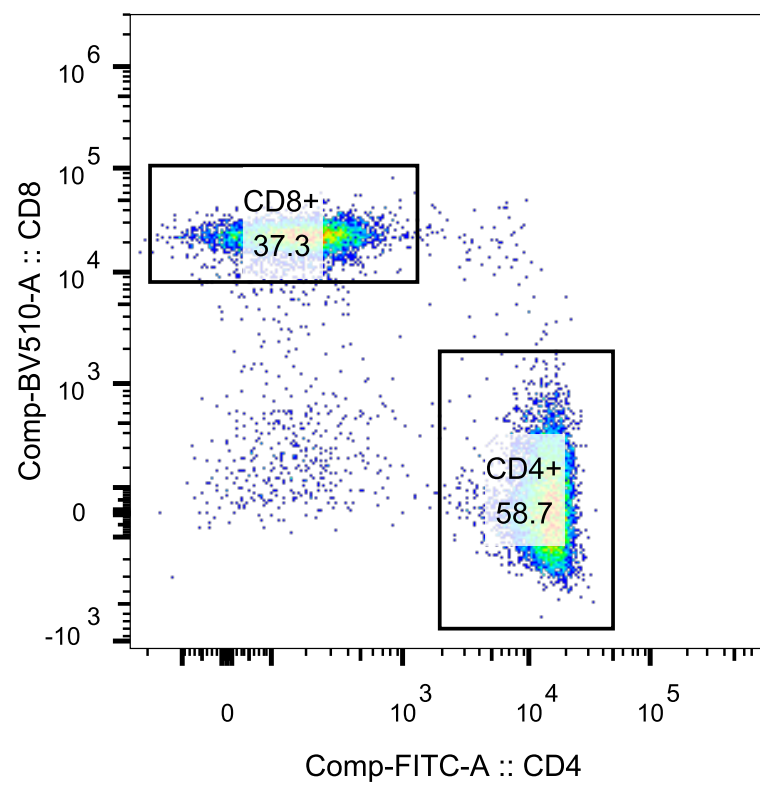

Supplement: Supplementary file 1 — Appendix S1 [file CAM4-12-3237-s004.pdf]
